# piRNAs and PIWI Proteins as Diagnostic and Prognostic Markers of Genitourinary Cancers

**DOI:** 10.3390/biom12020186

**Published:** 2022-01-22

**Authors:** Karolina Hanusek, Sławomir Poletajew, Piotr Kryst, Agnieszka Piekiełko-Witkowska, Joanna Bogusławska

**Affiliations:** 1 Centre of Postgraduate Medical Education, Department of Biochemistry and Molecular Biology, 01-813 Warsaw, Poland; karolina.hanusek@cmkp.edu.pl; 2 Centre of Postgraduate Medical Education, II Department of Urology, 01-813 Warsaw, Poland; slawomir.poletajew@cmkp.edu.pl (S.P.); piotr.kryst@cmkp.edu.pl (P.K.)

**Keywords:** genitourinary cancers, renal cancer, penile cancer, testicular cancer, bladder cancer, prostate cancer, PIWI proteins, PIWI-interacting RNAs, piRNAs, diagnosis

## Abstract

piRNAs (PIWI-interacting RNAs) are small non-coding RNAs capable of regulation of transposon and gene expression. piRNAs utilise multiple mechanisms to affect gene expression, which makes them potentially more powerful regulators than microRNAs. The mechanisms by which piRNAs regulate transposon and gene expression include DNA methylation, histone modifications, and mRNA degradation. Genitourinary cancers (GC) are a large group of neoplasms that differ by their incidence, clinical course, biology, and prognosis for patients. Regardless of the GC type, metastatic disease remains a key therapeutic challenge, largely affecting patients’ survival rates. Recent studies indicate that piRNAs could serve as potentially useful biomarkers allowing for early cancer detection and therapeutic interventions at the stage of non-advanced tumour, improving patient’s outcomes. Furthermore, studies in prostate cancer show that piRNAs contribute to cancer progression by affecting key oncogenic pathways such as PI3K/AKT. Here, we discuss recent findings on biogenesis, mechanisms of action and the role of piRNAs and the associated PIWI proteins in GC. We also present tools that may be useful for studies on the functioning of piRNAs in cancers.

## 1. Introduction

PIWI-interacting RNAs (piRNAs) are short (24–31 nt) ribonucleic acids belonging to the large family of non-coding RNAs (ncRNAs). Similarly to miRNAs, piRNAs are involved in the regulation of gene expression. However, they differ by length, number of encoding genes, biogenesis, as well as the range of regulatory actions (Table 1). piRNAs utilize a plethora of mechanisms affecting transposon and gene expression, including DNA methylation, histone modifications, and mRNA degradation.

piRNAs were discovered 20 years ago during studies on *Drosophila* [1]. Since then, their existence has been confirmed in multiple vertebrate and invertebrate species. Although the first studies in fly and mice were carried out in germline cells, later analyses demonstrated that piRNAs are expressed and function in somatic cells [2,3]. Mice studies revealed that compared with germline cell piRNAs, somatic piRNAs are shorter and show tissue-specific expression patterns, suggesting different roles played by both types of ncRNAs [4]. Multiple action modes of piRNAs enable them to act as crucial regulators of cell functioning. Remarkably, piRNAs show differences in expression, as well as genomic origin in tumour and normal cells [2], suggesting their cancer-specific roles. Indeed, recently published studies demonstrated that piRNAs can affect expression of oncogenes and tumour suppressors, contributing to cancer development and progression [5].

Genitourinary cancers (GC) are a large group of neoplasms, consisting 25% of all cancer types. They include common malignancies such as bladder and prostate cancer, as well as rare tumours such as penile cancer. The prognoses for GC patients largely differ, depending on cancer type and disease stage. Regardless of clinical differences between GC types, advanced, metastatic disease is always a therapeutic challenge and substantially worsens patients’ prognoses. Hence, early diagnosis, at the stage when tumour is localized, remains the best solution to improve cancer outcomes. This is best illustrated by the case of prostate cancer, the second most common malignancy in men. Introduction of PSA testing enabled early diagnosis and reduced prostate cancer mortality by nearly 50% [6]. However, for most GC types, clinically useful biomarkers allowing for early disease diagnosis are lacking. Similar difficulties are associated with detection of cancer recurrence. Remarkably, piRNAs offer several advantages as potential GC biomarkers due to their stability and easy detection in tumour tissues and plasma/serum [7,8]. Moreover, recent studies have demonstrated that piRNA expression profiles correlate with stage of disease and tumour aggressiveness, making them potentially useful prognostic biomarkers. Here, we comprehensively discuss the biogenesis and functioning of piRNAs in the context of genitourinary cancers.

## 2. Diagnosis and Prognosis of Genitourinary Cancers

Genitourinary cancers account for the fourth part of all cancers. They differ in biology, histology and hence clinical management and prognosis. Similarly, clinical diagnosis is usually associated with different clinical scenario. While most renal tumours are nowadays diagnosed incidentally at low stage with imaging, bladder tumours are still diagnosed in the face of haematuria, which can be associated with advanced disease. Prostate cancer is now usually diagnosed at an early stage thanks to active screening with PSA testing among men at risk, while once diagnosed, many cases do not need any treatment. Regarding rare penile and testicular cancers, they can be relatively easily diagnosed by self-examination, however, they both are still associated with a shame and fear of delaying medical intervention. As can be seen in these cases, difficulty in deciding to visit a doctor is universal and it concerns young men with testicle mass, as well as elderly men with penile lesions. Regarding prognostic factors in genitourinary cancers, they are not universal and are mainly based on pathological features, namely, cancer stage, cancer grade, variant histology, lymphovascular invasion.

### 2.1. Renal Cancer

There are more than 92,000 new cases of renal cancer in Europe yearly [9,10]. Renal cancer originating from the epithelium lining renal tubules is defined as renal cell carcinoma (RCC) and accounts for 90% of renal tumour cases. Main subtypes of RCC are clear cell (ccRCC), papillary (pRCC), and chromophobe (chRCC) [11]. They differ in genetics, a nephron part they originate from, histology, morphology, as well as prognosis. What stays universal are diagnostic tools and clinical management.

As stated above, nowadays, most renal cancer cases are diagnosed incidentally, at the stage of asymptomatic and clinically silent tumours [12]. This is because abdominal ultrasound became almost universally available and frequently used tool for imaging, also in patients without any abdominal symptoms. Imaging modality of choice to describe renal masses, as well as clinical staging of renal cancer is computed tomography (CT), alternatively magnetic resonance imaging (MRI) in complex cases or in the presence of contraindications to CT [13,14]. While surgical treatment consisting in removal of a tumour (partial nephrectomy) or a kidney (radical nephrectomy) remains the standard of care, the popularity of alternative management is increasing. In cases of small renal masses of <4 cm in elderly patients, renal tumour biopsy followed by active surveillance or tumour cryo- or radiofrequency ablation is more and more frequently adopted [15,16]. In cases of advanced cancer, systemic therapies with angiogenesis-targeted agents and/or checkpoint inhibitors offer high response rate and relatively long-term survival. In selected patients, also cytoreductive nephrectomy improves survival [17,18].

From a clinical point of view, prognosis in renal cancer patients is based mainly on disease stage at the time of initial treatment and histological features. In general, it is estimated that 49% of RCC patients are alive 5 years after diagnosis [19,20]. At the same time, the risk of death in patients with nodal and distant metastases is 16- and 33-fold higher, respectively, when compared to patients with localized tumours of <7 cm [21]. Moreover, the location of metastases does also play a prognostic role, with bone, liver, pleura, and brain metastases being associated with the shortest survival, not exceeding 18 months [22]. Another significant prognostic factor is histological type [23,24]. The longest overall survival rates are reported for pRCC followed by chRCC and ccRCC [25]. Rare carcinoma of the collecting ducts is associated with significantly shorter survival [26]. Finally, there are numerous proposed RNA and protein biomarkers, including multiple-biomarker models [27]. However, they are not used routinely in clinical practice [28].

There are universal nomograms predicting survival of patients with localized renal tumours. They are routinely used postoperatively to predict the risk of relapse and personalize follow-up. These models incorporate tumour stage, grade, and patient performance status or age [29,30]. There are also specific prognostic factors associated with histology type, i.e., tumour necrosis in ccRCC cases [25], tumour thrombus in pRCC cases [31], or fat invasion and sarcomatoid differentiation in chRCC [32]. In cases of metastatic renal cancer, there are well-established biochemical and clinical prognostic factors, including serum calcium, lactate dehydrogenase, haemoglobin levels, neutrophil and platelet count, patient general status according to Karnofsky performance status, and time interval from diagnosis to treatment. They are incorporated into two universal prognostic models, namely, MSKCC (Memorial Sloan Kettering Cancer Center) and IMDC (International Metastatic Renal Cancer Database Consortium) models [33,34]. They both stratify an individual patient into one of three risk categories. Median overall survival in favourable-risk patients reaches 30–43 months, while in poor-risk patients, it does not exceed 5–8 months [33,34]. In addition to clinical factors, there is an increasing body of evidence behind molecular prognostic factors, including genomic, transcriptomic, and proteomic factors [35].

### 2.2. Bladder Cancer

Bladder cancer is the most common malignancy of the urinary tract. It is also the seventh the most common cancer in men [36]. From a clinical perspective, there are two types of bladder cancer, namely, non-muscle-invasive bladder cancer (NMIBC) and muscle-invasive bladder cancer (MIBC). They differ substantially not only in stage but also in genetics, biology, clinical management, and prognosis [37]. The clear majority of cases are diagnosed in face of painless haematuria. Cystoscopy is the preferred method to confirm the bladder tumour presence, while MRI and CT are used for local and distant staging, respectively. An initial step in the treatment is transurethral resection of the tumour, enabling staging and guiding further treatment. Standard histological prognostic factors are not only stage and grade, but also the presence of variant histology and lymphovascular invasion [38,39,40].

In NMIBC cases, treatment consists of endoscopic resection followed by intravesical chemo- or immunotherapy. Despite adjuvant therapies, the risk of recurrence is relatively high. The most important prognostic factors are presented in EORTC and CUETO nomograms. They include tumour stage, grade, presence of carcinoma in situ foci, number of tumours, tumour size, previous recurrence rate, age, gender [41,42]. Depending on these parameters, 5-year risk of progression is >1% in low-risk group and 40–44% in high-risk group [43]. The risk of disease progression is strongly associated with disease stage and cancer grade [44].

In MIBC cases, the standard of care in non-metastatic patients is neoadjuvant chemotherapy and radical cystectomy [45]. Aggressive ablative surgery is followed by urinary diversion. The surgery itself is associated with 2–8% mortality rate and 50% risk of surgical complications [46,47,48,49,50]. Negative postoperative prognostic factors include advanced disease stage, especially extravesical extension and lymph node involvement [51], presence of the tumour in prostatic urethra [52], and high neutrophil-to-lymphocyte ratio [53]. Moreover, there are five molecular bladder cancer subtypes with different genetic profiles and clinical prognoses, grouped into two main molecular groups, namely luminal and basal-squamous [54]. Although they are well described, they are still not routinely used in clinical practice.

In metastatic bladder cancer systemic chemotherapy, cisplatin-based whenever possible is the standard of care. Unfortunately, the response rates, as well as survival rates, are unsatisfactory. Median survival in metastatic patients treated with first-line chemotherapy is 12–14 months [37]. Classical poor prognostic factors are Karnofsky performance status <80% and presence of visceral metastases [55]. Newer models incorporate additional data on location and extent of metastases, as well as laboratory findings, including leukocyte count, albumin, and haemoglobin levels [56,57]. Checkpoint inhibitors are a new treatment option in a second-line or in patients unfit for cisplatin. In these cases, the response rate is 21–29% and median survival is 8–16 months depending on clinical indications and tested agent [58]. Among prognostic factors of treatment outcome, one can consider PD-L1 expression, tumour mutation burden, tumour-infiltrating lymphocytes and gene expression profiles and others [59].

### 2.3. Prostate Cancer

Prostate cancer is the second most common malignancy in men [60]. The diagnosis of prostate cancer has been revolutionized with the implementation of PSA testing and multiparametric MRI. The first did improve diagnosis and increase the rate of cases diagnosed at early stage [61], the latter did decrease the rate of clinically insignificant cancers [62]. Apart from physical examination, PSA and MRI, there is a great number of blood-, urine- or tissue-based biomarkers that assess the risk of prostate cancer [63]. They are routinely used to decide whether an individual patient should undergo a prostate biopsy. Clinical diagnosis of prostate cancer is based on prostate biopsy, usually triggered by suspicious MRI image in patients with abnormal digital rectal examination or elevated PSA [64]. Apart from cancer grade, there are several histological poor prognostic factors that can be diagnosed at this point, including lymphovascular invasion, extraprostatic extension, presence of cribriform pattern or intraductal carcinoma [65,66]. Moreover, there are multiple-protein or multiple-gene-based commercial tests of significant prognostic value [67].

Treatment choice is guided by several clinical factors, including life expectancy, disease stage, cancer grade, PSA level, patient’s preference, etc. In cases of localized disease, standard treatment options are active surveillance, watchful waiting, radical prostatectomy, or radiation therapy with or without adjuvant androgen deprivation therapy (ADT). The risk of post-treatment recurrence depends mainly on local cancer stage, tumour grade and PSA level [68] detailed models incorporate also nodal status, percentage of positive cores at biopsy and age [69]. In patients treated surgically, a status of surgical margin also plays a prognostic role [70]. The risk of progression can also be assessed with dedicated molecular tools, i.e., the Decipher gene signature [71].

Biochemical recurrence of prostate cancer after radical treatment affects 27–53% of patients [64]. They can benefit from salvage surgery or radiotherapy, as well as androgen deprivation therapy. However, intervention is not needed in many cases, as clinical manifestation of disease recurrence may never occur [72]. Poor prognostic factors in these patients include PSA doubling time <1 year, high-grade cancer, time interval to biochemical recurrence <18 months [73].

In metastatic patients, standard treatment is ADT and systemic docetaxel chemotherapy. Median survival of 42 months is reported [74], however, it varies significantly between individual patients. There are several clinical prognostic factors, including PSA response at seven months of ADT [75], volume of disease [76], presence of visceral metastases, cancer grade, patient performance status, and others [77,78].

### 2.4. Testicular Cancer

Testicular cancer typically affects men in their third or fourth life decade [79]. Palpable testicle mass can be easily confirmed by the ultrasound, while distant staging requires chest and abdominal CT [80]. Standard treatment is radical surgery, namely orchiectomy, which in most cases is followed by systemic chemotherapy or radiation therapy [79]. There are numerous prognostic factors in patients with testicular cancer. The postoperative risk of relapse is increased in patients with tumour size of >4 cm and invasion of the rete testis in cases of seminoma and lymphovascular invasion in peri-tumoral tissue in cases of non-seminoma tumours [81,82,83].

In patients with metastatic disease, cytoreductive surgery followed by systemic chemotherapy is a standard of care. According to typical protocol, patients receive bleomycin, etoposide, and cisplatin [79]. The prognosis in metastatic patients can be assessed based on a system developed by The International Germ Cell Cancer Collaborative Group, which includes location of metastases and serum levels of three testicular cancer biomarkers: alpha-fetoprotein, human chorionic gonadotrophin, lactate dehydrogenase. Patients are stratified into three risk groups with different prognoses. Five-year progression-free survival is 89 and 90% in the good-prognosis group and decreases to 54% and 79% in the poor-prognosis group among patients with non-seminoma and seminoma tumours, respectively [84,85].

### 2.5. Penile Cancer

Penile cancer is a rare tumour, that usually arises from inner prepuce or glans penis. A visible and/or palpable lesion requires a biopsy, which is followed by treatment. Clinical staging bases on palpation of both groins to exclude metastatic inguinal lymph nodes, in positive cases followed by computed tomography imaging to exclude distant metastases [86]. Depending on disease stage, management can be local treatment with topical agents, CO_2_ or ND:Yag laser, surgery (circumcision, glans resurfacing, glansectomy, partial or radical penectomy) or radiation therapy [87]. Together with local surgery, patients at high risk of nodal metastases can undergo invasive inguinal nodal staging, while those with clinically enlarged lymph nodes warrant inguinal lymphadenectomy [88]. Penile cancer is a chemosensitive neoplasm. Systemic treatment with cisplatin and 5-fluorouracil with or without taxane is an option of adjuvant treatment in operated patients with lymph node metastases, as well as it remains a standard palliative therapy for patients with metastatic disease [89].

Among prognostic factors, one should list disease stage, pathological subtype, and cancer grade. While the clear majority of cases are squamous cell carcinomas, basaloid and sarcomatoid variants are associated with significantly higher cancer specific mortality [86]. Moreover, the presence of lymphovascular invasion, perineural invasion, and depth of invasion are other factors of prognostic significance [90,91].

## 3. Biogenesis of piRNAs and the Mechanism of Their Action in Mammalian Cells

Historically, the first piRNAs were identified in 2001 during the study of the role of small RNAs in spermatogenesis in *Drosophila* testes [92]. Initially, they were described as rasiRNAs (Repeat Associated RNAs) and their name was changed to piRNAs after the discovery of their interactions with PIWI proteins [93,94]. The first mammalian piRNAs were identified in 2006 in mouse testes during seeking of PIWI-interacting ncRNAs by four independent research teams [1,95,96,97]. The biogenesis and function of piRNAs in *Drosophila* and other lower organisms have been fully described elsewhere [98,99]. Here, we focus on mammalian piRNAs.

Deep-sequencing followed by computational analyses revealed that piRNA-coding sequences are grouped in clusters at the defined loci (≤200 kb) throughout the genome. In the mouse genome, over 96% of piRNAs are clustered at a few hundred sites [100,101]. The location of mouse piRNA clusters is mainly euchromatic, while in humans, most piRNAs clusters are located in intergeneric regions, and only a few in transposons, suggesting that transposable elements silencing is not a major role for piRNAs [102].

Transcription of piRNAs is catalyzed by Polymerase II (RNAPol II) which produces long single-stranded RNA precursors instead of stem-loop precursors (Table 1) and resembles canonical mRNA transcription. The piRNAs clusters harbour repressive histone 3 lysine 9 trimethylation (H3K9me3) marks at transcription start sites (TSS), which play a pivotal role in recruiting transcription initiation factors [103]. In mouse spermatocytes during the pachytene stage of meiosis transcription of these clusters is also regulated by transcription factors such as A-MYB. Similarly, in humans, A-MYB was shown to coordinate the transcription of about 55% of pachytene piRNAs and about 30% of genes encoding piRNAs biogenesis proteins [104].

The exact mechanism of pre-piRNA export to the cytoplasm still needs to be elucidated. In mice, piRNA precursors are bound by Maelstrom (MAEL), a conserved HMG box domain protein with RNA-binding activity, which facilitates their export to the cytoplasm [105].

Biogenesis of piRNAs is a complex process, classically divided into two collaborating routes. The first stage, synthesis of primary piRNAs, starts with transcription of piRNA-coding sequences in the nucleus, followed by posttranscriptional modifications leading to the maturation of piRNAs [106,107]. Primary piRNAs are generated from long single-stranded precursors of piRNAs by endonuclease MitoPLD/Zucchini (Zuc), with the following resolving of their secondary structure by the RNA helicase MOV10L1. Then, the precursors are processed by Zuc anchored to the outer membrane of mitochondria (OMM) into 25–40 nt intermediates. [108,109,110,111]. Although precise mechanisms and factors that determine Zuc cleavage still require further investigation, it was reported that PIWI proteins participate in this process. PIWI binds to the long piRNA precursor transcripts and determines the site of their endonucleolytic cleavage, which defines the characteristic length of mature piRNAs [108]. Loading of piRNAs precursors into PIWI requires Heat shock protein (Hsp 90) with cofactor-Shutdown (Shu) [112]. All piRNAs produced by Zuc cleavage revealed bias for uridine at their 5′end (1U bias) [113,114,115]. However, this feature of piRNAs is a result of PIWI PAZ domain preferential binding of piRNA intermediates beginning with U and probably is not connected with the selectivity of Zuc cleavage [116].

Finally, the 3′ ends of piRNAs are formed by Zuc in a process termed phasing or inch worming, by cleavages in Ping-Pong cycle, or by exonucleolytic trimming. Fragmentation of long piRNA precursor bound by PIWI proteins catalyzed by Zuc in 5′-3′ direction, leads to the production of a subset of tail-to-head, phased piRNA precursor [106]. Exonucleolytic trimming in 3′-to-5′ direction by TDRKH in mouse shortens a longer precursor formed by Zuc-mediated cleavage to produce mature piRNAs of the final length. The length of the trimmed, mature piRNAs depends on the specific PIWI protein to which the piRNAs was bound [111,117,118,119].

The last step in the maturation of piRNAs is the methylation of the 2′ hydrosyl at 3′ end of piRNAs by the *S*-adenosylmethionine-dependent methyltransferase HENMT1 in mouse which increases the stability of piRNAs [120,121].

Secondary piRNAs are produced by the Ping-Pong cycle also referred to as the amplification loop [122]. In mice, the Ping-Pong mechanism of piRNAs biogenesis is mainly processed by MILI and MIWI2, while in humans by HILI1 and HIWI2 [5]. This mechanism is triggered by pre-existing, maternal piRNAs or Zuc-mediated piRNAs generated from transposon regulatory regions of heterochromatin. These piRNAs loaded into MILI are cut between 10 nt and 11 nt at their 5′ ends, which results in piRNAs intermediates containing an adenine residues. Next, these intermediates are processed by MIWIL2 and their 3′ end is trimmed [123,124]. 

### 3.1. The Mechanism of Regulation of Genes and Transposons Expression via piRNA/PIWI Proteins

Initial piRNA studies were focused mainly on the mechanisms of piRNAs-dependent regulation of transposons expression. Nowadays, it is also known that piRNAs interact with the PIWI proteins and form a piRISC complex, which participates in transcriptional or posttranscriptional genes silencing (TGS and PTGS, respectively) or transcriptional or posttranscriptional activation (TGA and PTGA, respectively). piRNAs are also involved in posttranslational regulation of proteins stability.

### 3.2. Transposon Silencing

The most important and best-understood function of piRNAs is silencing of transposable elements (TEs). Tes are able to move from one location to another through cutting and reintegration mechanism. This leads to inhibition of gene expression or genomic DNA rearrangements (such as deletion, duplication, or inversion), and consequently may cause genome instability [125]. Therefore, silencing of transposons is crucial for maintaining genome integrity. The key role of piRNAs/PIWI proteins in this process was confirmed in the experiments with MILI/MIWI2 knockout mice. It was shown that loss of these proteins leads to increased TEs activity [126].

The expression of transposons can be inhibited by transcriptional and post-transcriptional mechanisms. In mice, transcriptional piRNA-mediated silencing of TEs is mainly accomplished by methylation of DNA. Binding of piRNA-MIWI2 complex recruits TDRD9, DNMT3L, DNMT3a, and DNMT3a2 proteins which catalyse DNA methylation [127]. Recently, it was demonstrated that interaction of MIWI2 with SPOCD1 protein is required for methylation and silencing of TEs [128].

In contrast, posttranscriptional inhibition occurs in the cytoplasm and is mediated by MIWI and MILI in mice, as well as PIWIL1 and PIWIL2 in humans, These PIWI-piRNA complexes, by binding to transposons transcripts govern their degradations through the “Ping-Pong“ mechanism [129].

### 3.3. Transcriptional Silencing or Activation of Genes Expression (TGS or TGA) 

PIWI/piRNA complex participate not only in TEs inhibition but also in the regulation of genes expression, in a mechanism similar to that observed for TEs. There are many examples of TGS in mammals’ somatic cells. For instance, in monocytes, modification of H3K9me in the promoter region of CD1A leads to the binding of HP1a and inhibition of this transcript expression. Methylation of H3K9 is induced by PIWIL4 interacting with SUV39H1 or SETDB1 proteins [130]. The role of piRNAs in the regulation of histones and DNA methylation was described for the CDKN2B gene in leukaemias. piR_014637 and piR_011186 stimulated K3K9 and H3K27 methylation and CpG methylation in the promoter region of CDKN2B through interaction with DNMT1, HMTs Suv39H1, and EZH2 [131]. piRNA-mediated regulation of DNA methylation resulting in downregulation of CREB2 expression was in turn, described in Aplasia neurons [132]. In breast cancer, piR_021285 inhibited ARHGAP11A expression through stimulation of methylation at CpG sites within the 5′UTR/first exon of these transcripts. ARHGAP11A is a known proapoptotic regulator, thus, a decrease in its expression results in inhibition of apoptosis [133]. It was also shown that piRNA_823 enhances global DNA methylation in multiple myeloma (MM) through induction of DNMT3A and DNMT3B methyltransferases, which leads to inhibition of p16INK4A (tumour suppressor) expression [134]. Moreover, in prostate cancer (see Section 5.3), PIWIL4/piR_31470 complex participates in silencing of GSTP1 expression through stimulation of DNA methylation which promotes cancer progression [135].

Interestingly, piRNAs-mediated regulation of histones modifications can also lead to activation of gene expression. In particular, He et al. demonstrated in breast cancer that piR_sno75 derived from GAS5 (a tumour-suppressive lncRNA) upregulates the expression of TRAIL (tumour necrosis factor (TNF)-related apoptosis-inducing ligand) through simultaneous stimulation of H3K4 methylation and H3K27 demethylation. In this regulation, PIWIL1/4 proteins interact with WDR5 which subsequently recruit MLL3 and UTX proteins [136].

The above-mentioned studies illustrate the diversity of action of different piRNA/PIWI complexes in the regulation of genes expression through chromatin modification.

### 3.4. Posttranscriptional Silencing of Genes Expression (PTGS) via mRNA Degradation

Cytoplasmic piRNAs may also inhibit the expression of genes through direct mRNA binding followed by PIWI-mediated transcript degradation. This mechanism is similar to that of miRNAs. piRNAs target lncRNAs (long non-coding RNAs), pseudogenes, and mRNAs. In this interaction, the complementary base pairing between 2–11 nt at the 5′-end of piRNAs and 3′UTR of transcripts is essential for RNA degradation [120]. Such regulation was presented, inter alia, by Peng et al., who found that piR_55490 by binding to 3′UTR of mTOR mRNA, stimulates its degradation. In lung cancer, downregulation of this piRNA was observed, which results in mTOR overexpression and activation of cancer cells proliferation rates [137]. Similarly, piR_30188 inhibits expression of miR-367 sponge–lncRNA OIP5-AS1. A decrease in this piRNA expression observed in glioma malignant cells caused upregulation in OIP-AS1. This leads to the sponging of miR-367 and overexpression of its target gene, TRAF4, which stimulates cancer progression [138]. Interestingly, piRNA/PIWI binding sites were also identified in 5′UTRs of transcripts [139]. Importantly, piRNA/PIWI complex is also able to facilitate RNA decay by binding CCR4-NOT (carbon catabolite-repressed 4-negative on TATA-less) as well as SMG (Smaug) proteins and forming piRISC complex, which promotes its imperfect base-pairing with RNA and results in its degradation via miRNA-like mechanism [140].

piRNA/PIWI complexes induce degradation of RNAs by its deadenylation. In mouse elongating spermatids, piRISC complex interacts with CAF1 deadenylase, which stimulates deadenylation and decay of numerous RNAs, leading to nuclear condensation and cytoplasmic exclusion resulting in the formation of spermatozoa [141]. An example of such regulation in humans is downregulation of IL-4 (Interleukin-4) by piR_30840 in T lymphocytes. This piRNA in complex with PIWIL4 and Ago4 binds to the 3′UTR of IL-4 via sequence complementarity which induces degradation of IL-4 pre-mRNA through recruitment of Trf-Air2-Mtr4 polyadenylation complex (TRAMP) and shortening of poly-A tails of this transcript. This in turn leads to decay of IL-4 pre-mRNA via nuclear exosomes and inhibition of Th2 CD4+ T-lymphocytes development [142].

### 3.5. piRNA/PIWIs-Mediated Regulation of Posttranslational Modifications

piRNA/PIWI complexes regulate posttranslational modifications such as phosphorylation and ubiquitination. For instance, piR_823 binds HSF1 (transcription factor of heat shock proteins, HSPs) which stimulates its phosphorylation at Ser326 and increases its transcriptional activity, which results in induction of HSPs expression and stimulates proliferation rates of colorectal cancer cells (CRC) [143]. Moreover, interaction of piR_54265, another CRC-expressed piRNA, with PIWILI2 results in formation of PIWIL2/STAT3/phosphorylated-SRC (pSRC) complex which stimulates STAT3 phosphorylation. In turn, this results in activation of proliferation, metastasis, and chemoresistance of cancer cells [144]. Additionally, Li et al. demonstrated that PIWIL1 inhibits polymerization of microtubules and induces proliferation, migration, and invasion of tumour cells via upregulation of STMN1 (stathmin). PIWIL1 inhibited degradation of STMN1 by preventing its ubiquitination by ligase RLIM as well as reduced stathmin phosphorylation through suppression of the interaction between STMN1 and CaMKII (calmodulin-dependent protein kinase II) [145].

## 4. Methodology of piRNA Analyses

The rapidly growing interest in piRNAs contributed to the development of the methodology of their research, which seems to be similar to that used for microRNAs. snRNA-seq and microarrays are the most often used assays for piRNAs identification and quantification [146,147]. There are also some studies describing the use of crosslinking immunoprecipitation sequencing (CLIP-seq) and RNA immunoprecipitation sequencing (RIP-seq) for detection of piRNAs in complex with PIWI/Argonaute proteins [148,149]. These methods generate a large amount of data, therefore, computational programs for their analysis were required. Recently, numerous bioinformatic methods were developed for piRNAs identification, analysis of their functions, and searching for homologous piRNAs and piRNA clusters (Table 2). In order to experimentally verify the results of large-scale studies northern blotting, in situ hybridization as well as qRT-PCR (quantitative reverse transcription-polymerase chain reaction) are performed [150,151,152,153]. RNA22 version 2.0 (https://cm.jefferson.edu/rna22/; [154] last accessed on 19 January 2022), IntaRNA version 5.0.0 (http://rna.informatik.uni-freiburg.de/IntaRNA/Input.jsp; [155] last accessed on 19 January 2022) and RNAhybrid (https://bibiserv.cebitec.uni-bielefeld.de/rnahybrid; [156] last accessed on 19 January 2022) algorithms are used to identify piRNAs targets, considering strict base pairing within 2–11 nt at 5′-end of piRNAs, as well as less rigorous base pairing within 12–21 nt and target mRNAs [120]. To confirm interactions of piRNAs with target mRNAs, cells are transfected with piRNA mimics or antisense inhibitors, then luciferase assays, Western blots, and qRT-PCRs are performed [157]. Additionally, in vitro, functional tests such as proliferation, migration, and invasion assays are done after transfection cells with piRNA mimics and inhibitors [144]. Moreover, these synthetic molecules are also used for in vivo studies in animal xenograft models to study their effects on tumour growth [158]. Interestingly, recently molecular beacons are used for visualization of piRNAs biogenesis and direct interaction with their target genes; this method can be of great importance in cancer therapy [139].

## 5. The Role of piRNAs in GC

### 5.1. Renal Cancer

In renal cancers, several studies have described the association of PIWI proteins and piRNAs expression with RCC diagnosis, prognosis, and treatment (Table 3). The expression of PIWIL1-4 genes was analysed in paired ccRCC-normal tissue samples, and higher expression of PIWIL4 in tumour tissue was observed. Moreover, ccRCC occurred earlier in patients with higher expression of PIWIL1 gene [179]. In contrast, downregulated expression of PIWIL1, PIWIL2, and PIWIL4 genes was observed in high-stage RCC tumour samples and was associated with worse overall survival (OS) of patients [180]. Immunohistochemical analysis revealed that PIWIL1 expression correlated with higher tumour grade and clinical staging, distant metastasis, and shorter CSS (cancer-specific survival), as well as high pre-operative CRP levels [181].

With regards to piRNAs, it was demonstrated that downregulation of mitochondrial piR_34536 and piR_51810 correlated with shorter disease-free survival (DFS) and OS [182], while decreased expression of another piRNA: piR_823 was related to a longer DFS of ccRCC patients [152]. Interestingly, the expression of piR_823 was significantly higher in blood, serum, and urine of ccRCC patients, but did not correlate with clinicopathological parameters of tumour [152]. Moreover, Busch et al. revealed reduced expression of piR_38756, piR_57125, and piR_30924 in non-metastatic primary RCC tumours compared with normal renal tissue. Interestingly, the expression of piR_57125 was decreased while piR_30924 and piR_38756 were increased in metastatic RCC samples and bone metastases. Additionally, the expression of piR_30924 and piR_38756 positively correlated with tumour grade and clinical stage [153]. Shorter CSS and a higher RCC stage and probability of metastasis were also associated with upregulated expression of piR_32051/piR_39894/piR_43607 cluster [183]. Considering the above, PIWI proteins and piRNAs appear to be useful biomarkers for RCC detection and treatment, however, their utility needs to be validated in independent studies.

**Table 3:** Disturbances of piRNAs and PIWIL expression in genitourinary cancers.

Cancer	Molecules	Sample Type	Expression	Function	References
Renal cancer	PIWIL1	Tissues	Up/Down	Prognosis biomarker	[179,180,181]
	PIWIL2	Tissues	Down	Prognosis biomarker	[180]
	PIWIL4	Tissues	Up/Down	Prognosis biomarker	[179,180]
	piR_32051	Tissues	Up	Diagnostic biomarker	[183]
	piR_39894	Tissues	Up	Diagnostic biomarker	
	piR_43607	Tissues	Up	Prognosis biomarker	
	piR_30924	Tissues	Down	Prognosis biomarker	[153]
	piR_38756	Tissues	Down	Prognosis biomarker	
	piR_57125	Tissues	Down	Prognosis biomarker	
	piR_34536	Tissues/serum	Down	Prognosis biomarker	[182]
	piR_51810	Tissues/serum	Down	Prognosis biomarker	
	piR_823	Tissues/serum/urine	Down	Prognosis biomarker	[152,180]
Penile cancer	piR_35280	Tissues	Down	Diagnostic biomarker	[184]
	piR_43773	Tissues	Down	Diagnostic biomarker	
Testicular cancer	PIWIL1	Tissues	Up (only in seminomas)	Prognosis biomarker	[185]
		Tissues	Down	Diagnostic biomarker	[186]
	PIWIL2	Tissues	Up (only in seminomas)	Prognosis biomarker/role in the regulation of apoptosis and proliferation	[187]
		Tissues	Down	Diagnostic biomarker	[186]
	PIWIL4	Tissues	Down	Diagnostic biomarker	[186]
	DQ598918	Tissues	Down	Diagnostic biomarker	[186]
	DQ589977	Tissues	Down	Diagnostic biomarker	
	DQ601609	Tissues	Down	Diagnostic biomarker	
	piR_004172	Tissues	Down	Diagnostic biomarker	[188]
	piR_006113	Tissues	Down	Diagnostic biomarker	
	piR_007509	Tissues	Down	Diagnostic biomarker	
Bladder cancer	PIWIL1	Tissues		Prognostic biomarkers	[189]
	PIWIL2	Tissues		Prognostic biomarker	[189,190]
	piR_DQ594040 (piRABC)	Tissues	Down	Diagnostic tool/target gene: TNFSF4	[191]
Prostate cancer	PIWIL2	Serum	Up	Prognostic biomarker	[192]
	PIWIL2	Tissues/Cell lines	Up	Prognosis biomarker/potential treatment target	[193]
	piR_31470	Tissues	Up	Diagnostic biomarker/target gene: GSTP	[135]
	piR_DQ722010	Mouse tissues	Down	Promotion of prostate hyperplasia activation PI3K/AKT signalling	[194]
	piR_000627	Tissues	-	Prognosis biomarker	[195]
	piR_005553	Tissues	-	Prognosis biomarker	
	piR_019346	Tissues	-	Prognosis biomarker	
	piR_000312	Tissues	-	Prognosis biomarker	
	piR_011079	Tissues	-	Prognosis biomarker	
	piR_012366	Tissues	-	Prognosis biomarker	
	piR_011389	Tissues	-	Prognosis biomarker	
	piR_19004	Tissues	Up	Diagnostic biomarker	[196]
	piR_2878	Tissues	Up	Diagnostic biomarker	
	piR_19166	Tissues	Down	Diagnostic biomarker	
	piR_349843	Urine	UP	Diagnostic biomarker	[197]
	piR_382289	Urine	UP	Diagnostic biomarker	
	piR_158533	Urine	UP	Diagnostic biomarker	
	piR_002468	Urine	UP	Diagnostic biomarker	
	piR_001773	Tissues	UP	Potential molecular target	[198]
	piR_017184	Tissues	UP	Potential molecular target	

### 5.2. Bladder Cancer

Up to date, only three studies described the role of PIWI proteins/piRNAs in bladder cancer (Table 3). A study based on evaluation of cytoplasmic or nuclear PIWIL2 expression by immunohistochemistry (IHC) in 202 tumour samples of chemotherapy-treated bladder cancer (BCa) demonstrated that a combination of weak cytoplasmic and lack of nuclear PIWIL2 expression correlates with poor prognosis for BCa patients [190]. In contrast, another study performed by the same team demonstrated that muscle-invasive bladder cancer (MIBC) patients with high PIWIL2 as well as PIWIL1 expression had poorer disease-specific survival (DSS) and recurrence-free survival (RFS). The authors indicated that one of the possible reasons for this difference could be the fact that the previous study was carried out on chemotherapy-treated patients, whereas in the second study, only 28% of patients received chemotherapy [189].

piRNAs expression was evaluated in only one study which identified 106 upregulated and 91 downregulated piRNAs in BCa. The top downregulated piRNA was piR_DQ594040 (piRABC). Its overexpression inhibited cell proliferation and colony formation, and promoted cell apoptosis in BCa cell line, indicating that piRABC contributes to cancerous transformation of the bladder [191].

### 5.3. Prostate Cancer

Several studies describe the disturbed expression of piRNAs and PIWI proteins as well as the association of piRNAs with PC diagnosis, prognosis, and treatment (Table 3). In particular, it was found that PIWIL2 expression was associated with the Gleason score and the TNM (Tumour Node Metastasis) stage. Knocked-down PIWIL2 decreased invasion and migration of prostate cancer-derived PC 3 cell line. This was associated with reduced expression of matrix metalloproteinase 9 (MMP9) and EMT markers. These results suggest that PIWIL2 could be a therapeutic target for the treatment of prostate cancer [193]. Furthermore, it was found that serum PIWIL2 was higher in patients with high ISUP grade groups indicating its association with more invasive and aggressive cancers. These data suggest that serum PIWIL2 could be a prognostic biomarker for advanced PCa stages [192].

Regarding the piRNAs, analysis of transcriptomic data from >100 PCa samples identified three piRNAs (piR_000627, piR_005553, and piR_019346) associated with PCa biochemical recurrence, suggesting their potential utility as prognostic biomarkers. Additionally, four piRNAs (piR_011389, piR_000312, piR_011079, and piR_012366) showed differential expression between PCa ISUP grade groups 2 and 3. This indicates that the four piRNAs could be helpful in clinical classification of PCa tumours [195]. Additionally, overexpression of piR_001773 and piR_017184 was found in prostate tumours compared with the adjacent normal tissue. Both of these piRNAs posttranscriptionally regulate PCDH9 tumour suppressor which acts as a negative regulator of PI3K/AKT pathway. Downregulation of PCDH9 by piR_001773 and piR_017184 in PCa cells resulted in an increase in AKT phosphorylation and activity. In contrast, downregulation of piR_001773 and piR_017184 inhibited tumour growth both in vitro and in vivo. This suggests that piR_001773 and piR_017184 represent potential molecular targets for PCa therapy [198]. Another study identified upregulation of piR_19004 and piR_2878 and down-regulation of piR_19166 in PCa tissues compared with normal prostate tissues. Cortactin (CTTN) was found as a direct target of piR_19166. Transfection of PCa cells by piR_19166 suppressed migration and metastasis via CTTN/matrix metalloproteinases (MMPs) pathway in PCa cells. Authors propose that piR_19166, through regulation of the oncogene CTTN, inhibits migration and distant metastasis of prostate cancer cells and may represent a new marker of diagnosis and treatment for PCa patients in early stages [196].

piRNAs may also be promising non-invasive PCa biomarkers. Specifically, increased expressions of piR_349843, piR_382289, piR_158533, and piR_002468 was detected in urinary EVs (extracellular vesicles) of PCa patients when compared with the control group. The analyses of AUC of these four piRs, as well as their combinations, indicated their specificity for PCa [197].

Interesting data were provided regarding the molecular effects of piRNA dysregulation in PCa. Studies performed on a murine model of chemically induced prostatic hyperplasia revealed that downregulation of piRNA_DQ722010 induces activation of PI3K/AKT pathway by upregulating the expression of PIK3R3. Increased PI3K/AKT signalling promotes prostate hyperplasia and is one of the most commonly activated pathways in prostate cancer [194]. Another mechanism involved in piRNA-mediated induction of cancer initiation and progression was proposed for piR_31470 of which expression is increased in PCa tumours. It was found that piR_31470 attenuates the expression of glutathione *S*-transferase π 1 (GSTP1), which plays a pivotal role in protecting cells from damage induced by oxidative stress. Specifically, piR_31470 complexed with PIWIL4 induces hypermethylation of GSTP1 promoter region, leading to downregulation of its expression. This in turn leads to increased oxidative stress and DNA damage in human prostate epithelial cells. These data are reflected in patient observations as GSTP1 hypermethylation is a common event during the initiation of prostate carcinogenesis [135].

### 5.4. Testicular Cancer

Despite the well-established role of PIWIL and piRNAs in spermatogenesis and function of normal human testis [102,199,200,201,202], little is known about their involvement in testicular cancer development and progression. Qiao et al. demonstrated that the expression of HIWI (PIWIL1) depends on differentiation stage of germ cells during spermatogenesis. Moreover, expression of PIWIL1 and PIWIL2 was upregulated in seminomas, but not in non-seminoma tumours [185]. Additionally, ROC curve analysis indicated that PIWIL2 could be used as a biomarker of testicular germ cell tumours (TGCTs) [203].

In contrast, Hempfling’s team found a decrease in PIWIL1 and PIWIL2 expression in samples from TGCTs biopsies compared with normal testis [204]. Downregulation of PIWIL1, PIWIL2, PIWIL4, and TDRD1 genes expression due to hypermethylation of their promoters was also observed in primary TGCTs (both seminomas and nonseminomas) in comparison to normal testicular tissues [186]. Decreased expression of these proteins attenuates expression of piRNAs (DQ598918, DQ589977, and DQ601609) and DNA methylation of LINE1 transposon [186].

PIWIL2 is expressed in TGCTs as two isoforms, 80 kDa (PL2L80A) and 60 kDa (PL2L60A), with predominant expression of the latter. This isoform lacks the catalytic centre (responsible for PIWIL2 slicing activity) and N-terminal domain (necessary for the formation of complexes with piRNAs) [205]. Interestingly, the expression of the short PIWIL2 isoform was high in undifferentiated seminomas and decreased in tumours with a greater degree of differentiation. Thus, PL2L60A might be used as a biomarker to distinguish between seminomas and non-seminoma tumours [206]. Moreover, it was shown that silencing of PL2L60A in TERA1 (TGCTs-derived) cell line results in posttranscriptional inhibition of transposons [207].

Gainetdinov et al. analysed the biogenesis and function of piRNAs in germ cells in four types of tissue samples: healthy adult testes, germ cells adjacent to TGCTs, GCNIS (germ cell neoplasia in situ) cells, and TGCT cells. They revealed downregulated expression of genes associated with piRNAs biogenesis in TGCTs and GCNIS samples, compared to control tissue [207]. Global inhibition of piRNAs expression (particularly piR_004172, piR_006113, and piR_007509) in TGCTs compared to the normal testis was also reported by Rounge and coworkers by sequencing of small RNAs [188].

### 5.5. Penile Cancer

Penile cancer is rarely diagnosed, therefore, its molecular basis, including the role of PIWI proteins and piRNAs, is poorly understood. In the work published in 2015, Zhang and coworkers showed that piR_49145, piR_34811, piR_49143, piR_36041, piR_33880, piR_49144, piR_35280, piR_43773, piR_33081, and piR_36173 are the most abundant piRNAs in penile cancers and matched adjacent normal penile tissues. piR_43773 and piR_35280 were downregulated in cancer samples compared with non-tumour controls, which may indicate their diagnostic potential (Table 3) [184].

## 6. Conclusions and Future Perspectives

piRNAs are novel small non-coding RNAs that show promise as potential diagnostic and prognostic biomarkers of genitourinary cancers. The studies on the role of PIWI proteins and piRNAs in GC are still in their infancy and multiple questions remained to be answered. In particular, there is a need for large-scale analyses of piRNAs expressed in tumour tissues as well as sera of patients with rare tumours, such as penile cancer. On the other hand, contradicting data regarding piRNA expression in more common cancers such as testicular cancer remain to be verified. Furthermore, a surprisingly small number of studies have been performed on bladder cancer, which is the fourth most common cancer in men. For all GC types, already obtained data on piRNAs as potential diagnostic and prognostic biomarkers require validation on independent cohorts of patients. Apart from their diagnostic potential, piRNAs emerge as crucial regulators of molecular pathways during cancer development and progression. Unfortunately, the data on the functional consequences of piRNA dysregulation in GC are scarce. Studies performed on prostate cancer cells indicate that piRNAs and PIWI proteins regulate key oncogenic pathways involving PI3K/AKT and EMT regulators. However, to the best of our knowledge, no such studies were published so far for renal, penile, testicular, and bladder cancers. Undoubtedly, future studies are needed to further explore the clinical and molecular significance of piRNAs and PIWI proteins in genitourinary cancers.

## Figures and Tables

**Table 1:** Differences between miRNAs and piRNAs.

Characteristics	miRNAs	piRNAs
Length	18–25 nt	24–31 nt
Genomic localization	Non-coding and coding gene regions	Transposable elements, non-coding, and coding gene regions
Precursor	Double-stranded, hairpin RNA	Single-stranded RNA
Modification of 3′ end	None	2′-*O*-methylation
Mechanism of biosynthesis	Dependent on Dicer	Independent of Dicer
Function	Induction of mRNA degradation and inhibition of translation	mRNA and transposons repression, DNA methylation, histone modifications, protein interaction
Targets	Protein coding genes	Transposons and protein coding genes

**Table 2:** Bioinformatic tools for analysis of piRNAs sequences and prediction of its targets.

Database	Content	Organisms	Website (Last Accessed on 4 December 2021)	Reference
piRDisease v1.0	Web service providing experimentally verified data about role of 4796 piRNAs in 28 diseases	Humans	http://www.piwirna2disease.org/index.php	[159]
piRNAQuest	Web service providing information about piRNAs’ clusters, annotation, significant motifs, and expression of piRNAs in different tissues and developmental stages.	Human, mouse, and rat	http://bicresources.jcbose.ac.in/zhumur/pirnaquest/	[160]
piRBase V3.0	Web service giving information about piRNAs function and annotation.	Human, mouse, rat, *D. melanogaster*, *C. elegans*, zebrafish, chicken, silkworm, cow, pig, horse	http://bigdata.ibp.ac.cn/piRBase/index.php	[161]
IsopiRBank	Web service providing information about isoforms of piRNAs, their annotation, target prediction, and enrichment analysis.	Human, mouse, *D. rerio* and *D. melanogaster*	http://mcg.ustc.edu.cn/bsc/isopir/index.html	[162]
piRNA cluster database	Web service presenting extensive data on piRNAs clusters in various species, tissue, and developmental stages.	Many species from Actinopterygii, Amphibia, Arechnidia, Ares, Bivalvia, Gastropoda, Insecta, Reptilia and Mammalia classes including human, mouse, or rat.	http://www.smallrnagroup-mainz.de/piRNAclusterDB.html	[163]
piRNN	Freely available user downloadable program for identification of piRNAs from small RNA sequencing data.	Human, rat, *C. elegans*, *D. melanogaster*.	https://github.com/bioinfolabmu/piRNN	[164]
IpiRId	Web service for prediction of piRNAs	Human, mouse, *D. melanogaster*	https://evryrna.ibisc.univ-evry.fr/evryrna/IpiRId/ipirid_home	[165]
piRPred	Web service for prediction of piRNAs	Human, *D. melanogaster*	https://evryrna.ibisc.univ-evry.fr/evryrna/piRPred/home	[166]
piRNABank	Web service providing information about piRNAs annotations, piRNAs clusters and homologous piRNAs	Human, mouse, rat, *D. melanogaster*	http://pirnabank.ibab.ac.in/	[167]
piRNAdb	Web service presenting data on piRNAs alignments, tissue expression, clusters, target genes, and ontology terms	Human, mouse, rat, hamster	https://www.pirnadb.org/index	[168]
pirnaPRE	Web service providing potential targets mRNA for piRNAs	Mouse	http://www.regulatoryrna.org/software/piRNA/piRNA_target_mRNA/index.php	[169]
SEAweb	Web database for investigation of small RNA (miRNAs, piRNAs, snoRNAs, snRNAs, and siRNAs) and pathogens based on results of sRNA sequencing datasets analyzed with Oasis 2 pipelines	Human	https://sea.ims.bio/	[170]
sRNAPrimerDB	Comprehensive web service for design qPCR primers or probes for expression analysis of miRNAs, piRNAs, and siRNAs	Human, mouse	http://www.srnaprimerdb.com/	[171]
PingPongPro	Freely available user down-loadable program for identification of piRNAs which are amplified through the “ping-pong cycle” in piRNA-Seq data	Human, mouse, *C. elegans*, *D. rerio*, *D. melanogaster*	https://github.com/suhrig/pingpongpro	[172]
piClust	Web service identifying piRNAs clusters and transcripts from small RNA-seq data	Human, mouse, rat, chicken, honeybee, *Xenopus laevis*, zebrafish	http://epigenomics.snu.ac.kr/piclustweb/	[173]
2L-piRNA	Web service identifying piRNAs and their function	Mouse	http://bioinformatics.hitsz.edu.cn/2L-piRNA/server	[174]
piPipes	Freely available user down-loadable program for analysis piRNAs and other transposon-derived RNAs from high-throughput sequencing data	Human, mouse, *D. melanogaster*	https://github.com/bowhan/piPipes	[175]
proTRAC	Freely available user down-loadable program for prediction of piRNAs genomic cluster	Human, mouse, and diverse animal species	https://www.smallrnagroup.uni-mainz.de/	[176]
unitas	Freely available user down-loadable program for annotation of small RNAs including piRNAs	Human, mouse, and diverse animal species	https://www.smallrnagroup.uni-mainz.de/	[177]
PILFER	Freely available user down-loadable program for prediction clusters in piRNAs sequences	Human	https://github.com/rishavray/PILFER	[178]

## Data Availability

Not applicable.

## References

[1] Aravin A., Gaidatzis D., Pfeffer S., Lagos-Quintana M., Landgraf P., Iovino N., Morris P., Brownstein M.J., Kuramochi-Miyagawa S., Nakano T. (2006). A novel class of small RNAs bind to MILI protein in mouse testes. Nature.

[2] Martinez V.D., Vucic E.A., Thu K.L., Hubaux R., Enfield K.S., Pikor L.A., Becker-Santos D.D., Brown C.J., Lam S., Lam W.L. (2015). Unique somatic and malignant expression patterns implicate PIWI-interacting RNAs in cancer-type specific biology. Sci. Rep..

[3] Rizzo F., Hashim A., Marchese G., Ravo M., Tarallo R., Nassa G., Giurato G., Rinaldi A., Cordella A., Persico M. (2014). Timed regulation of P-element-induced wimpy testis-interacting RNA expression during rat liver regeneration. Hepatology.

[4] Ross R.J., Weiner M.M., Lin H. (2014). PIWI proteins and PIWI-interacting RNAs in the soma. Nature.

[5] Han Y.N., Li Y., Xia S.Q., Zhang Y.Y., Zheng J.H., Li W. (2017). PIWI Proteins and PIWI-Interacting RNA: Emerging Roles in Cancer. Cell Physiol. Biochem..

[6] Hugosson J., Carlsson S., Aus G., Bergdahl S., Khatami A., Lodding P., Pihl C.G., Stranne J., Holmberg E., Lilja H. (2010). Mortality results from the Goteborg randomised population-based prostate-cancer screening trial. Lancet Oncol..

[7] Qu A., Wang W., Yang Y., Zhang X., Dong Y., Zheng G., Wu Q., Zou M., Du L., Wang Y. (2019). A serum piRNA signature as promising non-invasive diagnostic and prognostic biomarkers for colorectal cancer. Cancer Manag Res..

[8] Yang X., Cheng Y., Lu Q., Wei J., Yang H., Gu M. (2015). Detection of stably expressed piRNAs in human blood. Int. J. Clin. Exp. Med..

[9] Ferlay J., Steliarova-Foucher E., Lortet-Tieulent J., Rosso S., Coebergh J.W., Comber H., Forman D., Bray F. (2013). Cancer incidence and mortality patterns in Europe: Estimates for 40 countries in 2012. Eur. J. Cancer.

[10] Capitanio U., Bensalah K., Bex A., Boorjian S.A., Bray F., Coleman J., Gore J.L., Sun M., Wood C., Russo P. (2019). Epidemiology of Renal Cell Carcinoma. Eur. Urol..

[11] Moch H., Cubilla A.L., Humphrey P.A., Reuter V.E., Ulbright T.M. (2016). The 2016 WHO Classification of Tumours of the Urinary System and Male Genital Organs-Part A: Renal, Penile, and Testicular Tumours. Eur. Urol..

[12] Novara G., Ficarra V., Antonelli A., Artibani W., Bertini R., Carini M., Cosciani Cunico S., Imbimbo C., Longo N., Martignoni G. (2010). Validation of the 2009 TNM version in a large multi-institutional cohort of patients treated for renal cell carcinoma: Are further improvements needed?. Eur. Urol..

[13] Putra L.G., Minor T.X., Bolton D.M., Appu S., Dowling C.R., Neerhut G.J. (2009). Improved assessment of renal lesions in pregnancy with magnetic resonance imaging. Urology.

[14] Kabala J.E., Gillatt D.A., Persad R.A., Penry J.B., Gingell J.C., Chadwick D. (1991). Magnetic resonance imaging in the staging of renal cell carcinoma. Br. J. Radiol..

[15] Uhlig J., Strauss A., Rucker G., Seif Amir Hosseini A., Lotz J., Trojan L., Kim H.S., Uhlig A. (2019). Partial nephrectomy versus ablative techniques for small renal masses: A systematic review and network meta-analysis. Eur. Radiol..

[16] Hu X., Shao Y.X., Wang Y., Yang Z.Q., Yang W.X., Li X. (2019). Partial nephrectomy versus ablative therapies for cT1a renal masses: A Systematic Review and meta-analysis. Eur. J. Surg. Oncol..

[17] Mejean A., Ravaud A., Thezenas S., Colas S., Beauval J.B., Bensalah K., Geoffrois L., Thiery-Vuillemin A., Cormier L., Lang H. (2018). Sunitinib Alone or after Nephrectomy in Metastatic Renal-Cell Carcinoma. N. Engl. J. Med..

[18] Bex A., Mulders P., Jewett M., Wagstaff J., van Thienen J.V., Blank C.U., van Velthoven R., Del Pilar Laguna M., Wood L., van Melick H.H.E. (2019). Comparison of Immediate vs Deferred Cytoreductive Nephrectomy in Patients With Synchronous Metastatic Renal Cell Carcinoma Receiving Sunitinib: The SURTIME Randomized Clinical Trial. JAMA Oncol..

[19] Li P., Wong Y.N., Armstrong K., Haas N., Subedi P., Davis-Cerone M., Doshi J.A. (2016). Survival among patients with advanced renal cell carcinoma in the pretargeted versus targeted therapy eras. Cancer Med..

[20] Wahlgren T., Harmenberg U., Sandstrom P., Lundstam S., Kowalski J., Jakobsson M., Sandin R., Ljungberg B. (2013). Treatment and overall survival in renal cell carcinoma: A Swedish population-based study (2000–2008). Br. J. Cancer.

[21] Keegan K.A., Schupp C.W., Chamie K., Hellenthal N.J., Evans C.P., Koppie T.M. (2012). Histopathology of surgically treated renal cell carcinoma: Survival differences by subtype and stage. J. Urol..

[22] Dudani S., Velasco G.d., Wells C., Gan C.L., Donskov F., Porta C., Fraccon A., Pasini F., Hansen A.R., Bjarnason G.A. (2020). Sites of metastasis and survival in metastatic renal cell carcinoma (mRCC): Results from the International mRCC Database Consortium (IMDC). J. Clin. Oncol..

[23] Cheville J.C., Lohse C.M., Zincke H., Weaver A.L., Blute M.L. (2003). Comparisons of outcome and prognostic features among histologic subtypes of renal cell carcinoma. Am. J. Surg. Pathol..

[24] Patard J.J., Leray E., Rioux-Leclercq N., Cindolo L., Ficarra V., Zisman A., De La Taille A., Tostain J., Artibani W., Abbou C.C. (2005). Prognostic value of histologic subtypes in renal cell carcinoma: A multicenter experience. J. Clin. Oncol..

[25] Leibovich B.C., Lohse C.M., Crispen P.L., Boorjian S.A., Thompson R.H., Blute M.L., Cheville J.C. (2010). Histological subtype is an independent predictor of outcome for patients with renal cell carcinoma. J. Urol..

[26] Abern M.R., Tsivian M., Polascik T.J., Coogan C.L. (2012). Characteristics and outcomes of tumors arising from the distal nephron. Urology.

[27] Petitprez F., Ayadi M., de Reynies A., Fridman W.H., Sautes-Fridman C., Job S. (2021). Review of Prognostic Expression Markers for Clear Cell Renal Cell Carcinoma. Front. Oncol..

[28] Raimondi A., Sepe P., Zattarin E., Mennitto A., Stellato M., Claps M., Guadalupi V., Verzoni E., de Braud F., Procopio G. (2020). Predictive Biomarkers of Response to Immunotherapy in Metastatic Renal Cell Cancer. Front. Oncol..

[29] Zisman A., Pantuck A.J., Wieder J., Chao D.H., Dorey F., Said J.W., deKernion J.B., Figlin R.A., Belldegrun A.S. (2002). Risk group assessment and clinical outcome algorithm to predict the natural history of patients with surgically resected renal cell carcinoma. J. Clin. Oncol..

[30] Buti S., Puligandla M., Bersanelli M., DiPaola R.S., Manola J., Taguchi S., Haas N.B. (2017). Validation of a new prognostic model to easily predict outcome in renal cell carcinoma: The GRANT score applied to the ASSURE trial population. Ann. Oncol..

[31] Klatte T., Gallagher K.M., Afferi L., Volpe A., Kroeger N., Ribback S., McNeill A., Riddick A.C.P., Armitage J.N., Aho T.F. (2019). The VENUSS prognostic model to predict disease recurrence following surgery for non-metastatic papillary renal cell carcinoma: Development and evaluation using the ASSURE prospective clinical trial cohort. BMC Med..

[32] Leibovich B.C., Lohse C.M., Cheville J.C., Zaid H.B., Boorjian S.A., Frank I., Thompson R.H., Parker W.P. (2018). Predicting Oncologic Outcomes in Renal Cell Carcinoma After Surgery. Eur. Urol..

[33] Motzer R.J., Bacik J., Murphy B.A., Russo P., Mazumdar M. (2002). Interferon-alfa as a comparative treatment for clinical trials of new therapies against advanced renal cell carcinoma. J. Clin. Oncol..

[34] Heng D.Y., Xie W., Regan M.M., Harshman L.C., Bjarnason G.A., Vaishampayan U.N., Mackenzie M., Wood L., Donskov F., Tan M.H. (2013). External validation and comparison with other models of the International Metastatic Renal-Cell Carcinoma Database Consortium prognostic model: A population-based study. Lancet Oncol..

[35] Gan C.L., Dudani S., Heng D.Y.C. (2020). Prognostic and Predictive Factors in Metastatic Renal Cell Carcinoma: Current Perspective and a Look Into the Future. Cancer J..

[36] Siegel R.L., Miller K.D., Fuchs H.E., Jemal A. (2021). Cancer Statistics, 2021. CA Cancer J. Clin..

[37] Babjuk M., Burger M., Capoun O., Cohen D., Comperat E.M., Dominguez Escrig J.L., Gontero P., Liedberg F., Masson-Lecomte A., Mostafid A.H. (2021). European Association of Urology Guidelines on Non-muscle-invasive Bladder Cancer (Ta, T1, and Carcinoma in Situ). Eur. Urol..

[38] Veskimae E., Espinos E.L., Bruins H.M., Yuan Y., Sylvester R., Kamat A.M., Shariat S.F., Witjes J.A., Comperat E.M. (2019). What Is the Prognostic and Clinical Importance of Urothelial and Nonurothelial Histological Variants of Bladder Cancer in Predicting Oncological Outcomes in Patients with Muscle-invasive and Metastatic Bladder Cancer? A European Association of Urology Muscle Invasive and Metastatic Bladder Cancer Guidelines Panel Systematic Review. Eur. Urol. Oncol..

[39] Mathieu R., Lucca I., Roupret M., Briganti A., Shariat S.F. (2016). The prognostic role of lymphovascular invasion in urothelial carcinoma of the bladder. Nat. Rev. Urol..

[40] Mari A., Kimura S., Foerster B., Abufaraj M., D’Andrea D., Gust K.M., Shariat S.F. (2018). A systematic review and meta-analysis of lymphovascular invasion in patients treated with radical cystectomy for bladder cancer. Urol. Oncol..

[41] Sylvester R.J., van der Meijden A.P., Oosterlinck W., Witjes J.A., Bouffioux C., Denis L., Newling D.W., Kurth K. (2006). Predicting recurrence and progression in individual patients with stage Ta T1 bladder cancer using EORTC risk tables: A combined analysis of 2596 patients from seven EORTC trials. Eur. Urol..

[42] Fernandez-Gomez J., Madero R., Solsona E., Unda M., Martinez-Pineiro L., Gonzalez M., Portillo J., Ojea A., Pertusa C., Rodriguez-Molina J. (2009). Predicting nonmuscle invasive bladder cancer recurrence and progression in patients treated with bacillus Calmette-Guerin: The CUETO scoring model. J. Urol..

[43] Sylvester R.J., Rodriguez O., Hernandez V., Turturica D., Bauerova L., Bruins H.M., Brundl J., van der Kwast T.H., Brisuda A., Rubio-Briones J. (2021). European Association of Urology (EAU) Prognostic Factor Risk Groups for Non-muscle-invasive Bladder Cancer (NMIBC) Incorporating the WHO 2004/2016 and WHO 1973 Classification Systems for Grade: An Update from the EAU NMIBC Guidelines Panel. Eur. Urol..

[44] Cambier S., Sylvester R.J., Collette L., Gontero P., Brausi M.A., van Andel G., Kirkels W.J., Silva F.C., Oosterlinck W., Prescott S. (2016). EORTC Nomograms and Risk Groups for Predicting Recurrence, Progression, and Disease-specific and Overall Survival in Non-Muscle-invasive Stage Ta-T1 Urothelial Bladder Cancer Patients Treated with 1–3 Years of Maintenance Bacillus Calmette-Guerin. Eur. Urol..

[45] Witjes J.A., Bruins H.M., Cathomas R., Comperat E.M., Cowan N.C., Gakis G., Hernandez V., Linares Espinos E., Lorch A., Neuzillet Y. (2021). European Association of Urology Guidelines on Muscle-invasive and Metastatic Bladder Cancer: Summary of the 2020 Guidelines. Eur. Urol..

[46] Stein J.P., Skinner D.G. (2006). Radical cystectomy for invasive bladder cancer: Long-term results of a standard procedure. World J. Urol..

[47] Hautmann R.E., de Petriconi R.C., Pfeiffer C., Volkmer B.G. (2012). Radical cystectomy for urothelial carcinoma of the bladder without neoadjuvant or adjuvant therapy: Long-term results in 1100 patients. Eur. Urol..

[48] Hautmann R.E., de Petriconi R.C., Volkmer B.G. (2010). Lessons learned from 1,000 neobladders: The 90-day complication rate. J. Urol..

[49] Nielsen M.E., Mallin K., Weaver M.A., Palis B., Stewart A., Winchester D.P., Milowsky M.I. (2014). Association of hospital volume with conditional 90-day mortality after cystectomy: An analysis of the National Cancer Data Base. BJU Int..

[50] Porter M.P., Gore J.L., Wright J.L. (2011). Hospital volume and 90-day mortality risk after radical cystectomy: A population-based cohort study. World J. Urol..

[51] Dutta R., Abdelhalim A., Martin J.W., Vernez S.L., Faltas B., Lotan Y., Youssef R.F. (2016). Effect of tumor location on survival in urinary bladder adenocarcinoma: A population-based analysis. Urol. Oncol..

[52] Moschini M., Soria F., Susani M., Korn S., Briganti A., Roupret M., Seitz C., Gust K., Haitel A., Montorsi F. (2017). Impact of the Level of Urothelial Carcinoma Involvement of the Prostate on Survival after Radical Cystectomy. Bladder Cancer.

[53] Wu S., Zhao X., Wang Y., Zhong Z., Zhang L., Cao J., Ai K., Xu R. (2018). Pretreatment Neutrophil-Lymphocyte Ratio as a Predictor in Bladder Cancer and Metastatic or Unresectable Urothelial Carcinoma Patients: A Pooled Analysis of Comparative Studies. Cell Physiol. Biochem..

[54] Robertson A.G., Kim J., Al-Ahmadie H., Bellmunt J., Guo G., Cherniack A.D., Hinoue T., Laird P.W., Hoadley K.A., Akbani R. (2017). Comprehensive Molecular Characterization of Muscle-Invasive Bladder Cancer. Cell.

[55] Bajorin D.F., Dodd P.M., Mazumdar M., Fazzari M., McCaffrey J.A., Scher H.I., Herr H., Higgins G., Boyle M.G. (1999). Long-term survival in metastatic transitional-cell carcinoma and prognostic factors predicting outcome of therapy. J. Clin. Oncol..

[56] Apolo A.B., Ostrovnaya I., Halabi S., Iasonos A., Philips G.K., Rosenberg J.E., Riches J., Small E.J., Milowsky M.I., Bajorin D.F. (2013). Prognostic model for predicting survival of patients with metastatic urothelial cancer treated with cisplatin-based chemotherapy. J. Natl. Cancer Inst..

[57] Galsky M.D., Moshier E., Krege S., Lin C.C., Hahn N., Ecke T., Sonpavde G., Godbold J., Oh W.K., Bamias A. (2013). Nomogram for predicting survival in patients with unresectable and/or metastatic urothelial cancer who are treated with cisplatin-based chemotherapy. Cancer.

[58] Ghatalia P., Zibelman M., Geynisman D.M., Plimack E. (2018). Approved checkpoint inhibitors in bladder cancer: Which drug should be used when?. Ther. Adv. Med. Oncol..

[59] Stuhler V., Maas J.M., Bochem J., da Costa I.A., Todenhofer T., Stenzl A., Bedke J. (2019). Molecular predictors of response to PD-1/PD-L1 inhibition in urothelial cancer. World J. Urol..

[60] Rawla P. (2019). Epidemiology of Prostate Cancer. World J. Oncol..

[61] Hayes J.H., Barry M.J. (2014). Screening for prostate cancer with the prostate-specific antigen test: A review of current evidence. JAMA.

[62] Drost F.H., Osses D.F., Nieboer D., Steyerberg E.W., Bangma C.H., Roobol M.J., Schoots I.G. (2019). Prostate MRI, with or without MRI-targeted biopsy, and systematic biopsy for detecting prostate cancer. Cochrane Database Syst. Rev..

[63] Zapala P., Dybowski B., Poletajew S., Radziszewski P. (2018). What Can Be Expected from Prostate Cancer Biomarkers A Clinical Perspective. Urol. Int..

[64] Mottet N., van den Bergh R.C.N., Briers E., Van den Broeck T., Cumberbatch M.G., De Santis M., Fanti S., Fossati N., Gandaglia G., Gillessen S. (2021). EAU-EANM-ESTRO-ESUR-SIOG Guidelines on Prostate Cancer-2020 Update. Part 1: Screening, Diagnosis, and Local Treatment with Curative Intent. Eur. Urol..

[65] Kweldam C.F., Kummerlin I.P., Nieboer D., Verhoef E.I., Steyerberg E.W., van der Kwast T.H., Roobol M.J., van Leenders G.J. (2016). Disease-specific survival of patients with invasive cribriform and intraductal prostate cancer at diagnostic biopsy. Mod. Pathol..

[66] Saeter T., Vlatkovic L., Waaler G., Servoll E., Nesland J.M., Axcrona K., Axcrona U. (2017). Intraductal Carcinoma of the Prostate on Diagnostic Needle Biopsy Predicts Prostate Cancer Mortality: A Population-Based Study. Prostate.

[67] Eggener S.E., Rumble R.B., Armstrong A.J., Morgan T.M., Crispino T., Cornford P., van der Kwast T., Grignon D.J., Rai A.J., Agarwal N. (2020). Molecular Biomarkers in Localized Prostate Cancer: ASCO Guideline. J. Clin. Oncol..

[68] Zelic R., Garmo H., Zugna D., Stattin P., Richiardi L., Akre O., Pettersson A. (2020). Predicting Prostate Cancer Death with Different Pretreatment Risk Stratification Tools: A Head-to-head Comparison in a Nationwide Cohort Study. Eur. Urol..

[69] Dess R.T., Suresh K., Zelefsky M.J., Freedland S.J., Mahal B.A., Cooperberg M.R., Davis B.J., Horwitz E.M., Terris M.K., Amling C.L. (2020). Development and Validation of a Clinical Prognostic Stage Group System for Nonmetastatic Prostate Cancer Using Disease-Specific Mortality Results From the International Staging Collaboration for Cancer of the Prostate. JAMA Oncol..

[70] Yossepowitch O., Briganti A., Eastham J.A., Epstein J., Graefen M., Montironi R., Touijer K. (2014). Positive surgical margins after radical prostatectomy: A systematic review and contemporary update. Eur. Urol..

[71] Spratt D.E., Yousefi K., Deheshi S., Ross A.E., Den R.B., Schaeffer E.M., Trock B.J., Zhang J., Glass A.G., Dicker A.P. (2017). Individual Patient-Level Meta-Analysis of the Performance of the Decipher Genomic Classifier in High-Risk Men After Prostatectomy to Predict Development of Metastatic Disease. J. Clin. Oncol..

[72] Pound C.R., Partin A.W., Eisenberger M.A., Chan D.W., Pearson J.D., Walsh P.C. (1999). Natural history of progression after PSA elevation following radical prostatectomy. JAMA.

[73] Tilki D., Preisser F., Graefen M., Huland H., Pompe R.S. (2019). External Validation of the European Association of Urology Biochemical Recurrence Risk Groups to Predict Metastasis and Mortality After Radical Prostatectomy in a European Cohort. Eur. Urol..

[74] James N.D., Spears M.R., Clarke N.W., Dearnaley D.P., De Bono J.S., Gale J., Hetherington J., Hoskin P.J., Jones R.J., Laing R. (2015). Survival with Newly Diagnosed Metastatic Prostate Cancer in the “Docetaxel Era”: Data from 917 Patients in the Control Arm of the STAMPEDE Trial (MRC PR08, CRUK/06/019). Eur. Urol..

[75] Harshman L.C., Chen Y.H., Liu G., Carducci M.A., Jarrard D., Dreicer R., Hahn N., Garcia J.A., Hussain M., Shevrin D. (2018). Seven-Month Prostate-Specific Antigen Is Prognostic in Metastatic Hormone-Sensitive Prostate Cancer Treated With Androgen Deprivation With or Without Docetaxel. J. Clin. Oncol..

[76] Gravis G., Boher J.M., Chen Y.H., Liu G., Fizazi K., Carducci M.A., Oudard S., Joly F., Jarrard D.M., Soulie M. (2018). Burden of Metastatic Castrate Naive Prostate Cancer Patients, to Identify Men More Likely to Benefit from Early Docetaxel: Further Analyses of CHAARTED and GETUG-AFU15 Studies. Eur. Urol..

[77] Gravis G., Boher J.M., Fizazi K., Joly F., Priou F., Marino P., Latorzeff I., Delva R., Krakowski I., Laguerre B. (2015). Prognostic Factors for Survival in Noncastrate Metastatic Prostate Cancer: Validation of the Glass Model and Development of a Novel Simplified Prognostic Model. Eur. Urol..

[78] Glass T.R., Tangen C.M., Crawford E.D., Thompson I. (2003). Metastatic carcinoma of the prostate: Identifying prognostic groups using recursive partitioning. J. Urol..

[79] Albers P., Albrecht W., Algaba F., Bokemeyer C., Cohn-Cedermark G., Fizazi K., Horwich A., Laguna M.P., Nicolai N., Oldenburg J. (2015). Guidelines on Testicular Cancer: 2015 Update. Eur. Urol..

[80] Pierorazio P.M., Cheaib J.G., Tema G., Patel H.D., Gupta M., Sharma R., Zhang A., Bass E.B. (2020). Performance Characteristics of Clinical Staging Modalities for Early Stage Testicular Germ Cell Tumors: A Systematic Review. J. Urol..

[81] Boormans J.L., Mayor de Castro J., Marconi L., Yuan Y., Laguna Pes M.P., Bokemeyer C., Nicolai N., Algaba F., Oldenburg J., Albers P. (2018). Testicular Tumour Size and Rete Testis Invasion as Prognostic Factors for the Risk of Relapse of Clinical Stage I Seminoma Testis Patients Under Surveillance: A Systematic Review by the Testicular Cancer Guidelines Panel. Eur. Urol..

[82] Zengerling F., Kunath F., Jensen K., Ruf C., Schmidt S., Spek A. (2018). Prognostic factors for tumor recurrence in patients with clinical stage I seminoma undergoing surveillance—A systematic review. Urol. Oncol..

[83] Blok J.M., Pluim I., Daugaard G., Wagner T., Jozwiak K., Wilthagen E.A., Looijenga L.H.J., Meijer R.P., Bosch J., Horenblas S. (2020). Lymphovascular invasion and presence of embryonal carcinoma as risk factors for occult metastatic disease in clinical stage I nonseminomatous germ cell tumour: A systematic review and meta-analysis. BJU Int..

[84] Beyer J., Collette L., Daugaard G., Wit R.D., Tryakin A., Albany C., Stahl O., Fizazi K., Gietema J.A., Giorgi U.D. (2020). Prognostic factors in advanced seminoma: An analysis from the IGCCCG Update Consortium. J. Clin. Oncol..

[85] Gillessen S.C.L., Daugaard G., de Wit R., Tryakin A., Albany C., Stahl O., Fizazi K., Gietema J.A., De Giorgi U.F.F. (2019). 903O Redefining the IGCCCG classification in advanced non-seminoma. Ann. Oncol..

[86] Hakenberg O.W., Comperat E.M., Minhas S., Necchi A., Protzel C., Watkin N. (2015). EAU guidelines on penile cancer: 2014 update. Eur. Urol..

[87] Kulkarni M.Y.T., Bleicher G., Minhas S. (2018). Organ-sparing Treatment for Penile Cancer. Eur. Urol. Suppl..

[88] Watkin N. (2018). Lymph Node Staging in Clinically Negative Groin Nodes. Eur. Urol. Suppl..

[89] Necchi A. (2018). Systemic Therapy for Penile Cancer. Eur. Urol. Suppl..

[90] Winters B.R., Mossanen M., Holt S.K., Lin D.W., Wright J.L. (2016). Predictors of Nodal Upstaging in Clinical Node Negative Patients With Penile Carcinoma: A National Cancer Database Analysis. Urology.

[91] Cubilla A.L., Barreto J., Caballero C., Ayala G., Riveros M. (1993). Pathologic features of epidermoid carcinoma of the penis. A prospective study of 66 cases. Am. J. Surg. Pathol..

[92] Aravin A.A., Naumova N.M., Tulin A.V., Vagin V.V., Rozovsky Y.M., Gvozdev V.A. (2001). Double-stranded RNA-mediated silencing of genomic tandem repeats and transposable elements in the D. melanogaster germline. Curr. Biol..

[93] Aravin A.A., Lagos-Quintana M., Yalcin A., Zavolan M., Marks D., Snyder B., Gaasterland T., Meyer J., Tuschl T. (2003). The small RNA profile during Drosophila melanogaster development. Dev. Cell.

[94] Zamore P.D. (2010). Somatic piRNA biogenesis. EMBO J..

[95] Girard A., Sachidanandam R., Hannon G.J., Carmell M.A. (2006). A germline-specific class of small RNAs binds mammalian Piwi proteins. Nature.

[96] Grivna S.T., Beyret E., Wang Z., Lin H. (2006). A novel class of small RNAs in mouse spermatogenic cells. Genes Dev..

[97] Watanabe T., Takeda A., Tsukiyama T., Mise K., Okuno T., Sasaki H., Minami N., Imai H. (2006). Identification and characterization of two novel classes of small RNAs in the mouse germline: Retrotransposon-derived siRNAs in oocytes and germline small RNAs in testes. Genes Dev..

[98] Senti K.A., Brennecke J. (2010). The piRNA pathway: A fly’s perspective on the guardian of the genome. Trends Genet..

[99] Huang X., Fejes Toth K., Aravin A.A. (2017). piRNA Biogenesis in Drosophila melanogaster. Trends Genet..

[100] Chirn G.W., Rahman R., Sytnikova Y.A., Matts J.A., Zeng M., Gerlach D., Yu M., Berger B., Naramura M., Kile B.T. (2015). Conserved piRNA Expression from a Distinct Set of piRNA Cluster Loci in Eutherian Mammals. PLoS Genet..

[101] Assis R., Kondrashov A.S. (2009). Rapid repetitive element-mediated expansion of piRNA clusters in mammalian evolution. Proc. Natl. Acad. Sci. USA.

[102] Ha H., Song J., Wang S., Kapusta A., Feschotte C., Chen K.C., Xing J. (2014). A comprehensive analysis of piRNAs from adult human testis and their relationship with genes and mobile elements. BMC Genom..

[103] Wu X., Pan Y., Fang Y., Zhang J., Xie M., Yang F., Yu T., Ma P., Li W., Shu Y. (2020). The Biogenesis and Functions of piRNAs in Human Diseases. Mol. Ther. Nucleic Acids.

[104] Ozata D.M., Yu T., Mou H., Gainetdinov I., Colpan C., Cecchini K., Kaymaz Y., Wu P.H., Fan K., Kucukural A. (2020). Evolutionarily conserved pachytene piRNA loci are highly divergent among modern humans. Nat. Ecol. Evol..

[105] Castaneda J., Genzor P., van der Heijden G.W., Sarkeshik A., Yates J.R., Ingolia N.T., Bortvin A. (2014). Reduced pachytene piRNAs and translation underlie spermiogenic arrest in Maelstrom mutant mice. EMBO J..

[106] Pillai R.S., Chuma S. (2012). piRNAs and their involvement in male germline development in mice. Dev. Growth Differ..

[107] Fu Q., Wang P.J. (2014). Mammalian piRNAs: Biogenesis, function, and mysteries. Spermatogenesis.

[108] Izumi N., Shoji K., Suzuki Y., Katsuma S., Tomari Y. (2020). Zucchini consensus motifs determine the mechanism of pre-piRNA production. Nature.

[109] Zheng K., Xiol J., Reuter M., Eckardt S., Leu N.A., McLaughlin K.J., Stark A., Sachidanandam R., Pillai R.S., Wang P.J. (2010). Mouse MOV10L1 associates with Piwi proteins and is an essential component of the Piwi-interacting RNA (piRNA) pathway. Proc. Natl. Acad. Sci. USA.

[110] Beyret E., Liu N., Lin H. (2012). piRNA biogenesis during adult spermatogenesis in mice is independent of the ping-pong mechanism. Cell Res..

[111] Ding D., Liu J., Dong K., Melnick A.F., Latham K.E., Chen C. (2019). Mitochondrial membrane-based initial separation of MIWI and MILI functions during pachytene piRNA biogenesis. Nucleic Acids Res..

[112] Taborska E., Pasulka J., Malik R., Horvat F., Jenickova I., Jelic Matosevic Z., Svoboda P. (2019). Restricted and non-essential redundancy of RNAi and piRNA pathways in mouse oocytes. PLoS Genet..

[113] Perera B.P.U., Tsai Z.T., Colwell M.L., Jones T.R., Goodrich J.M., Wang K., Sartor M.A., Faulk C., Dolinoy D.C. (2019). Somatic expression of piRNA and associated machinery in the mouse identifies short, tissue-specific piRNA. Epigenetics.

[114] Wang W., Yoshikawa M., Han B.W., Izumi N., Tomari Y., Weng Z., Zamore P.D. (2014). The initial uridine of primary piRNAs does not create the tenth adenine that Is the hallmark of secondary piRNAs. Mol. Cell.

[115] Stein C.B., Genzor P., Mitra S., Elchert A.R., Ipsaro J.J., Benner L., Sobti S., Su Y., Hammell M., Joshua-Tor L. (2019). Decoding the 5’ nucleotide bias of PIWI-interacting RNAs. Nat. Commun..

[116] Simon B., Kirkpatrick J.P., Eckhardt S., Reuter M., Rocha E.A., Andrade-Navarro M.A., Sehr P., Pillai R.S., Carlomagno T. (2011). Recognition of 2’-O-methylated 3’-end of piRNA by the PAZ domain of a Piwi protein. Structure.

[117] Mohn F., Handler D., Brennecke J. (2015). Noncoding RNA. piRNA-guided slicing specifies transcripts for Zucchini-dependent, phased piRNA biogenesis. Science.

[118] Saxe J.P., Chen M., Zhao H., Lin H. (2013). Tdrkh is essential for spermatogenesis and participates in primary piRNA biogenesis in the germline. EMBO J..

[119] Chen C., Jin J., James D.A., Adams-Cioaba M.A., Park J.G., Guo Y., Tenaglia E., Xu C., Gish G., Min J. (2009). Mouse Piwi interactome identifies binding mechanism of Tdrkh Tudor domain to arginine methylated Miwi. Proc. Natl. Acad. Sci. USA.

[120] Goh W.S., Falciatori I., Tam O.H., Burgess R., Meikar O., Kotaja N., Hammell M., Hannon G.J. (2015). piRNA-directed cleavage of meiotic transcripts regulates spermatogenesis. Genes Dev..

[121] Lim S.L., Qu Z.P., Kortschak R.D., Lawrence D.M., Geoghegan J., Hempfling A.L., Bergmann M., Goodnow C.C., Ormandy C.J., Wong L. (2015). HENMT1 and piRNA Stability Are Required for Adult Male Germ Cell Transposon Repression and to Define the Spermatogenic Program in the Mouse. PLoS Genet..

[122] Weick E.M., Miska E.A. (2014). piRNAs: From biogenesis to function. Development.

[123] Parhad S.S., Theurkauf W.E. (2019). Rapid evolution and conserved function of the piRNA pathway. Open Biol..

[124] Li Y., Zhang Y., Liu M. (2021). Knockout Gene-Based Evidence for PIWI-Interacting RNA Pathway in Mammals. Front. Cell Dev. Biol..

[125] Ayarpadikannan S., Kim H.S. (2014). The impact of transposable elements in genome evolution and genetic instability and their implications in various diseases. Genom. Inform..

[126] Carmell M.A., Girard A., van de Kant H.J., Bourc’his D., Bestor T.H., de Rooij D.G., Hannon G.J. (2007). MIWI2 is essential for spermatogenesis and repression of transposons in the mouse male germline. Dev. Cell.

[127] Wang C., Lin H. (2021). Roles of piRNAs in transposon and pseudogene regulation of germline mRNAs and lncRNAs. Genome Biol..

[128] Zoch A., Auchynnikava T., Berrens R.V., Kabayama Y., Schopp T., Heep M., Vasiliauskaite L., Perez-Rico Y.A., Cook A.G., Shkumatava A. (2020). SPOCD1 is an essential executor of piRNA-directed de novo DNA methylation. Nature.

[129] Ozata D.M., Gainetdinov I., Zoch A., O’Carroll D., Zamore P.D. (2019). PIWI-interacting RNAs: Small RNAs with big functions. Nat. Rev. Genet..

[130] Zhang X., He X., Liu C., Liu J., Hu Q., Pan T., Duan X., Liu B., Zhang Y., Chen J. (2016). IL-4 Inhibits the Biogenesis of an Epigenetically Suppressive PIWI-Interacting RNA To Upregulate CD1a Molecules on Monocytes/Dendritic Cells. J. Immunol..

[131] Wu D., Fu H., Zhou H., Su J., Zhang F., Shen J. (2015). Effects of Novel ncRNA Molecules, p15-piRNAs, on the Methylation of DNA and Histone H3 of the CDKN2B Promoter Region in U937 Cells. J. Cell Biochem..

[132] Rajasethupathy P., Antonov I., Sheridan R., Frey S., Sander C., Tuschl T., Kandel E.R. (2012). A role for neuronal piRNAs in the epigenetic control of memory-related synaptic plasticity. Cell.

[133] Fu A., Jacobs D.I., Hoffman A.E., Zheng T., Zhu Y. (2015). PIWI-interacting RNA 021285 is involved in breast tumorigenesis possibly by remodeling the cancer epigenome. Carcinogenesis.

[134] Yan H., Wu Q.L., Sun C.Y., Ai L.S., Deng J., Zhang L., Chen L., Chu Z.B., Tang B., Wang K. (2015). piRNA-823 contributes to tumorigenesis by regulating de novo DNA methylation and angiogenesis in multiple myeloma. Leukemia.

[135] Zhang L., Meng X., Pan C., Qu F., Gan W., Xiang Z., Han X., Li D. (2020). piR-31470 epigenetically suppresses the expression of glutathione S-transferase pi 1 in prostate cancer via DNA methylation. Cell Signal..

[136] He X., Chen X., Zhang X., Duan X., Pan T., Hu Q., Zhang Y., Zhong F., Liu J., Zhang H. (2015). An Lnc RNA (GAS5)/SnoRNA-derived piRNA induces activation of TRAIL gene by site-specifically recruiting MLL/COMPASS-like complexes. Nucleic Acids Res..

[137] Peng L., Song L., Liu C., Lv X., Li X., Jie J., Zhao D., Li D. (2016). piR-55490 inhibits the growth of lung carcinoma by suppressing mTOR signaling. Tumour Biol..

[138] Ng K.W., Anderson C., Marshall E.A., Minatel B.C., Enfield K.S., Saprunoff H.L., Lam W.L., Martinez V.D. (2016). Piwi-interacting RNAs in cancer: Emerging functions and clinical utility. Mol. Cancer.

[139] Lee Y.J., Moon S.U., Park M.G., Jung W.Y., Park Y.K., Song S.K., Ryu J.G., Lee Y.S., Heo H.J., Gu H.N. (2016). Multiplex bioimaging of piRNA molecular pathway-regulated theragnostic effects in a single breast cancer cell using a piRNA molecular beacon. Biomaterials.

[140] Rouget C., Papin C., Boureux A., Meunier A.C., Franco B., Robine N., Lai E.C., Pelisson A., Simonelig M. (2010). Maternal mRNA deadenylation and decay by the piRNA pathway in the early Drosophila embryo. Nature.

[141] Gou L.T., Dai P., Yang J.H., Xue Y., Hu Y.P., Zhou Y., Kang J.Y., Wang X., Li H., Hua M.M. (2014). Pachytene piRNAs instruct massive mRNA elimination during late spermiogenesis. Cell Res..

[142] Zhong F., Zhou N., Wu K., Guo Y., Tan W., Zhang H., Zhang X., Geng G., Pan T., Luo H. (2015). A SnoRNA-derived piRNA interacts with human interleukin-4 pre-mRNA and induces its decay in nuclear exosomes. Nucleic Acids Res..

[143] Yin J., Jiang X.Y., Qi W., Ji C.G., Xie X.L., Zhang D.X., Cui Z.J., Wang C.K., Bai Y., Wang J. (2017). piR-823 contributes to colorectal tumorigenesis by enhancing the transcriptional activity of HSF1. Cancer Sci..

[144] Mai D., Ding P., Tan L., Zhang J., Pan Z., Bai R., Li C., Li M., Zhou Y., Tan W. (2018). PIWI-interacting RNA-54265 is oncogenic and a potential therapeutic target in colorectal adenocarcinoma. Theranostics.

[145] Li C., Zhou X., Chen J., Lu Y., Sun Q., Tao D., Hu W., Zheng X., Bian S., Liu Y. (2015). PIWIL1 destabilizes microtubule by suppressing phosphorylation at Ser16 and RLIM-mediated degradation of Stathmin1. Oncotarget.

[146] Yin J., Qi W., Ji C.G., Zhang D.X., Xie X.L., Ding Q., Jiang X.Y., Han J., Jiang H.Q. (2019). Small RNA sequencing revealed aberrant piRNA expression profiles in colorectal cancer. Oncol. Rep..

[147] Lin X., Xia Y., Hu D., Mao Q., Yu Z., Zhang H., Li C., Chen G., Liu F., Zhu W. (2019). Transcriptomewide piRNA profiling in human gastric cancer. Oncol. Rep..

[148] Dai P., Wang X., Gou L.T., Li Z.T., Wen Z., Chen Z.G., Hua M.M., Zhong A., Wang L., Su H. (2019). A Translation-Activating Function of MIWI/piRNA during Mouse Spermiogenesis. Cell.

[149] Shi S., Yang Z.Z., Liu S., Yang F., Lin H. (2020). PIWIL1 promotes gastric cancer via a piRNA-independent mechanism. Proc. Natl. Acad. Sci. USA.

[150] Phay M., Kim H.H., Yoo S. (2018). Analysis of piRNA-Like Small Non-coding RNAs Present in Axons of Adult Sensory Neurons. Mol. Neurobiol..

[151] Lu Y., Li C., Zhang K., Sun H., Tao D., Liu Y., Zhang S., Ma Y. (2010). Identification of piRNAs in Hela cells by massive parallel sequencing. BMB Rep..

[152] Iliev R., Fedorko M., Machackova T., Mlcochova H., Svoboda M., Pacik D., Dolezel J., Stanik M., Slaby O. (2016). Expression Levels of PIWI-interacting RNA, piR-823, Are Deregulated in Tumor Tissue, Blood Serum and Urine of Patients with Renal Cell Carcinoma. Anticancer Res..

[153] Busch J., Ralla B., Jung M., Wotschofsky Z., Trujillo-Arribas E., Schwabe P., Kilic E., Fendler A., Jung K. (2015). Piwi-interacting RNAs as novel prognostic markers in clear cell renal cell carcinomas. J. Exp. Clin. Cancer Res..

[154] Miranda K.C., Huynh T., Tay Y., Ang Y.S., Tam W.L., Thomson A.M., Lim B., Rigoutsos I. (2006). A pattern-based method for the identification of MicroRNA binding sites and their corresponding heteroduplexes. Cell.

[155] Mann M., Wright P.R., Backofen R. (2017). IntaRNA 2.0: Enhanced and customizable prediction of RNA-RNA interactions. Nucleic. Acids Res..

[156] Kruger J., Rehmsmeier M. (2006). RNAhybrid: microRNA target prediction easy, fast and flexible. Nucleic. Acids Res..

[157] Ding X., Li Y., Lu J., Zhao Q., Guo Y., Lu Z., Ma W., Liu P., Pestell R.G., Liang C. (2021). piRNA-823 Is Involved in Cancer Stem Cell Regulation Through Altering DNA Methylation in Association With Luminal Breast Cancer. Front. Cell Dev. Biol..

[158] Jacobs D.I., Qin Q., Fu A., Chen Z., Zhou J., Zhu Y. (2018). piRNA-8041 is downregulated in human glioblastoma and suppresses tumor growth in vitro and in vivo. Oncotarget.

[159] Muhammad A., Waheed R., Khan N.A., Jiang H., Song X. (2019). piRDisease v1.0: A manually curated database for piRNA associated diseases. Database.

[160] Sarkar A., Maji R.K., Saha S., Ghosh Z. (2014). piRNAQuest: Searching the piRNAome for silencers. BMC Genom..

[161] Wang J., Zhang P., Lu Y., Li Y., Zheng Y., Kan Y., Chen R., He S. (2019). piRBase: A comprehensive database of piRNA sequences. Nucleic Acids Res..

[162] Zhang H., Ali A., Gao J., Ban R., Jiang X., Zhang Y., Shi Q. (2018). IsopiRBank: A research resource for tracking piRNA isoforms. Database.

[163] Rosenkranz D. (2016). piRNA cluster database: A web resource for piRNA producing loci. Nucleic Acids Res..

[164] Wang K., Hoeksema J., Liang C. (2018). piRNN: Deep learning algorithm for piRNA prediction. PeerJ.

[165] Boucheham A., Sommard V., Zehraoui F., Boualem A., Batouche M., Bendahmane A., Israeli D., Tahi F. (2017). IpiRId: Integrative approach for piRNA prediction using genomic and epigenomic data. PLoS ONE.

[166] Brayet J., Zehraoui F., Jeanson-Leh L., Israeli D., Tahi F. (2014). Towards a piRNA prediction using multiple kernel fusion and support vector machine. Bioinformatics.

[167] Sai Lakshmi S., Agrawal S. (2008). piRNABank: A web resource on classified and clustered Piwi-interacting RNAs. Nucleic Acids Res..

[168] Piuco R., Galante P.A.F. (2021). piRNAdb: A piwi-interacting RNA database. bioRxiv.

[169] Yuan J., Zhang P., Cui Y., Wang J., Skogerbo G., Huang D.W., Chen R., He S. (2016). Computational identification of piRNA targets on mouse mRNAs. Bioinformatics.

[170] Rahman R.U., Liebhoff A.M., Bansal V., Fiosins M., Rajput A., Sattar A., Magruder D.S., Madan S., Sun T., Gautam A. (2020). SEAweb: The small RNA Expression Atlas web application. Nucleic Acids Res..

[171] Xie S., Zhu Q., Qu W., Xu Z., Liu X., Li X., Li S., Ma W., Miao Y., Zhang L. (2019). sRNAPrimerDB: Comprehensive primer design and search web service for small non-coding RNAs. Bioinformatics.

[172] Uhrig S., Klein H. (2019). PingPongPro: A tool for the detection of piRNA-mediated transposon-silencing in small RNA-Seq data. Bioinformatics.

[173] Jung I., Park J.C., Kim S. (2014). piClust: A density based piRNA clustering algorithm. Comput. Biol. Chem..

[174] Liu B., Yang F., Chou K.C. (2017). 2L-piRNA: A Two-Layer Ensemble Classifier for Identifying Piwi-Interacting RNAs and Their Function. Mol. Ther. Nucleic Acids.

[175] Han B.W., Wang W., Zamore P.D., Weng Z. (2015). piPipes: A set of pipelines for piRNA and transposon analysis via small RNA-seq, RNA-seq, degradome- and CAGE-seq, ChIP-seq and genomic DNA sequencing. Bioinformatics.

[176] Rosenkranz D., Zischler H. (2012). proTRACߞA software for probabilistic piRNA cluster detection, visualization and analysis. BMC Bioinformatics.

[177] Gebert D., Hewel C., Rosenkranz D. (2017). unitas: The universal tool for annotation of small RNAs. BMC Genom..

[178] Ray R., Pandey P. (2018). piRNA analysis framework from small RNA-Seq data by a novel cluster prediction tool PILFER. Genomics.

[179] Al-Janabi O., Wach S., Nolte E., Weigelt K., Rau T.T., Stohr C., Legal W., Schick S., Greither T., Hartmann A. (2014). Piwi-like 1 and 4 gene transcript levels are associated with clinicopathological parameters in renal cell carcinomas. Biochim. Biophys. Acta.

[180] Iliev R., Stanik M., Fedorko M., Poprach A., Vychytilova-Faltejskova P., Slaba K., Svoboda M., Fabian P., Pacik D., Dolezel J. (2016). Decreased expression levels of PIWIL1, PIWIL2, and PIWIL4 are associated with worse survival in renal cell carcinoma patients. OncoTargets Ther..

[181] Stohr C.G., Steffens S., Polifka I., Jung R., Kahlmeyer A., Ivanyi P., Weber F., Hartmann A., Wullich B., Wach S. (2019). Piwi-like 1 protein expression is a prognostic factor for renal cell carcinoma patients. Sci. Rep..

[182] Zhao C., Tolkach Y., Schmidt D., Toma M., Muders M.H., Kristiansen G., Muller S.C., Ellinger J. (2019). Mitochondrial PIWI-interacting RNAs are novel biomarkers for clear cell renal cell carcinoma. World J. Urol..

[183] Li Y., Wu X., Gao H., Jin J.M., Li A.X., Kim Y.S., Pal S.K., Nelson R.A., Lau C.M., Guo C. (2015). Piwi-Interacting RNAs (piRNAs) Are Dysregulated in Renal Cell Carcinoma and Associated with Tumor Metastasis and Cancer-Specific Survival. Mol. Med..

[184] Zhang L., Wei P., Shen X., Zhang Y., Xu B., Zhou J., Fan S., Hao Z., Shi H., Zhang X. (2015). MicroRNA Expression Profile in Penile Cancer Revealed by Next-Generation Small RNA Sequencing. PLoS ONE.

[185] Qiao D., Zeeman A.M., Deng W., Looijenga L.H., Lin H. (2002). Molecular characterization of hiwi, a human member of the piwi gene family whose overexpression is correlated to seminomas. Oncogene.

[186] Ferreira H.J., Heyn H., Garcia del Muro X., Vidal A., Larriba S., Munoz C., Villanueva A., Esteller M. (2014). Epigenetic loss of the PIWI/piRNA machinery in human testicular tumorigenesis. Epigenetics.

[187] Lee J.H., Schutte D., Wulf G., Fuzesi L., Radzun H.J., Schweyer S., Engel W., Nayernia K. (2006). Stem-cell protein Piwil2 is widely expressed in tumors and inhibits apoptosis through activation of Stat3/Bcl-XL pathway. Hum. Mol. Genet..

[188] Rounge T.B., Furu K., Skotheim R.I., Haugen T.B., Grotmol T., Enerly E. (2015). Profiling of the small RNA populations in human testicular germ cell tumors shows global loss of piRNAs. Mol. Cancer.

[189] Eckstein M., Jung R., Weigelt K., Sikic D., Stohr R., Geppert C., Agaimy A., Lieb V., Hartmann A., Wullich B. (2018). Piwi-like 1 and -2 protein expression levels are prognostic factors for muscle invasive urothelial bladder cancer patients. Sci. Rep..

[190] Taubert H., Wach S., Jung R., Pugia M., Keck B., Bertz S., Nolte E., Stoehr R., Lehmann J., Ohlmann C.H. (2015). Piwil 2 expression is correlated with disease-specific and progression-free survival of chemotherapy-treated bladder cancer patients. Mol. Med..

[191] Chu H., Hui G., Yuan L., Shi D., Wang Y., Du M., Zhong D., Ma L., Tong N., Qin C. (2015). Identification of novel piRNAs in bladder cancer. Cancer Lett..

[192] Tosun H., Demirtas A., Sonmez G., Tombul S.T., Akalin H., Ozkul Y. (2019). Can the expression level of PIWIL 2 gene be a serum marker for prostate cancer? A single-center prospective study. Turk. J. Urol..

[193] Yang Y., Zhang X., Song D., Wei J. (2015). Piwil2 modulates the invasion and metastasis of prostate cancer by regulating the expression of matrix metalloproteinase-9 and epithelial-mesenchymal transitions. Oncol. Lett..

[194] Han R., Zhang L., Gan W., Fu K., Jiang K., Ding J., Wu J., Han X., Li D. (2019). piRNA-DQ722010 contributes to prostate hyperplasia of the male offspring mice after the maternal exposed to microcystin-leucine arginine. Prostate.

[195] Zuo Y., Liang Y., Zhang J., Hao Y., Li M., Wen Z., Zhao Y. (2019). Transcriptome Analysis Identifies Piwi-Interacting RNAs as Prognostic Markers for Recurrence of Prostate Cancer. Front. Genet..

[196] Qi T., Cao H., Sun H., Feng H., Li N., Wang C., Wang L. (2020). piR-19166 inhibits migration and metastasis through CTTN/MMPs pathway in prostate carcinoma. Aging.

[197] Peng Q., Chiu P.K., Wong C.Y., Cheng C.K., Teoh J.Y., Ng C.F. (2021). Identification of piRNA Targets in Urinary Extracellular Vesicles for the Diagnosis of Prostate Cancer. Diagnostics.

[198] Zhang L., Meng X., Li D., Han X. (2020). piR-001773 and piR-017184 promote prostate cancer progression by interacting with PCDH9. Cell Signal..

[199] Keam S.P., Young P.E., McCorkindale A.L., Dang T.H., Clancy J.L., Humphreys D.T., Preiss T., Hutvagner G., Martin D.I., Cropley J.E. (2014). The human Piwi protein Hiwi2 associates with tRNA-derived piRNAs in somatic cells. Nucleic Acids Res..

[200] Yang Q., Hua J., Wang L., Xu B., Zhang H., Ye N., Zhang Z., Yu D., Cooke H.J., Zhang Y. (2013). MicroRNA and piRNA profiles in normal human testis detected by next generation sequencing. PLoS ONE.

[201] Cao C., Wen Y., Wang X., Fang N., Yuan S., Huang X. (2018). Testicular piRNA profile comparison between successful and unsuccessful micro-TESE retrieval in NOA patients. J. Assist. Reprod. Genet..

[202] Gainetdinov I., Skvortsova Y., Kondratieva S., Funikov S., Azhikina T. (2017). Two modes of targeting transposable elements by piRNA pathway in human testis. RNA.

[203] Gainetdinov I.V., Kondratieva S.A., Skvortsova Y.V., Zinovyeva M.V., Stukacheva E.A., Klimov A., Tryakin A.A., Azhikina T.L. (2016). Distinguishing epigenetic features of preneoplastic testis tissues adjacent to seminomas and nonseminomas. Oncotarget.

[204] Hempfling A.L., Lim S.L., Adelson D.L., Evans J., O’Connor A.E., Qu Z.P., Kliesch S., Weidner W., O’Bryan M.K., Bergmann M. (2017). Expression patterns of HENMT1 and PIWIL1 in human testis: Implications for transposon expression. Reproduction.

[205] Vagin V.V., Wohlschlegel J., Qu J., Jonsson Z., Huang X., Chuma S., Girard A., Sachidanandam R., Hannon G.J., Aravin A.A. (2009). Proteomic analysis of murine Piwi proteins reveals a role for arginine methylation in specifying interaction with Tudor family members. Genes Dev..

[206] Gainetdinov I.V., Skvortsova Y.V., Stukacheva E.A., Bychenko O.S., Kondratieva S.A., Zinovieva M.V., Azhikina T.L. (2014). Expression profiles of PIWIL2 short isoforms differ in testicular germ cell tumors of various differentiation subtypes. PLoS ONE.

[207] Gainetdinov I.V., Skvortsova Y.V., Kondratieva S.A., Klimov A., Tryakin A.A., Azhikina T.L. (2018). Assessment of piRNA biogenesis and function in testicular germ cell tumors and their precursor germ cell neoplasia in situ. BMC Cancer.

